# Loss of SIRT4 promotes the self-renewal of Breast Cancer Stem Cells

**DOI:** 10.7150/thno.44688

**Published:** 2020-07-25

**Authors:** Lutao Du, Xiaoyan Liu, Yidan Ren, Juan Li, Peilong Li, Qinlian Jiao, Peng Meng, Fang Wang, Yuli Wang, Yun-shan Wang, Chuanxin Wang

**Affiliations:** 1 Department of Clinical Laboratory, The Second Hospital, Cheeloo College of Medicine, Shandong University, 247 Beiyuan Street, Jinan, Shandong, 250033, China.; 2 International Biotechnology R&D Center, Shandong University School of Ocean, 180 Wenhua Xi Road, Weihai, Shandong 264209, China.; 3 The Medical Department of IVD Division, 3D Medicines, Inc., Pujiang Hi‑tech Park, Shanghai 201114, China.; 4 Institute of basic medicine, The Second Hospital, Cheeloo College of Medicine, Shandong University, 247 Beiyuan Street, Jinan, Shandong, 250033, China.

**Keywords:** SIRT4, SIRT1, breast cancer, cancer stemness, glutamine metabolism

## Abstract

**Rationale:** It has been proposed that cancer stem/progenitor cells (or tumor-initiating cells, TICs) account for breast cancer initiation and progression. Sirtuins are nicotinamide adenine dinucleotide (NAD+)-dependent class-III histone deacetylases and mediate various basic biological processes, including metabolic homeostasis. However, interplay and cross-regulation among the sirtuin family are not fully understood. As one of the least studied sirtuin family members, the mitochondrial sirtuin SIRT4 is a tumor suppressor gene in various cancers. However, its role in cancer stemness, as well as initiation and progression of breast cancer, remains unknown.

**Methods:** The expression of SIRT4 in breast cancer was analyzed using the TCGA breast cancer database and 3 GSEA data. Normal breast epithelial cells MCF10A and breast cancer cell lines MCF-7, MDA-MB-231, BT549, MDA-MB-468 were used to establish SIRT4 gene knockdown and corresponding overexpression cells. Identified MTT cytotoxicity assays, cell invasion and motility assay, sorting of SP, confocal immunofluorescence microscopy, mouse mammary stem cell analysis, glutamine and glucose production, clonogenic and sphere-formation assay, mass spectrometric metabolomics analysis and ChIP-seq to further explore SIRT4 biological role in breast cancer.

**Results:** We elucidated a novel role for SIRT4 in the negative regulation of mammary gland development and stemness, which is related to the mammary tumorigenesis. We also uncovered an inverse correlation between SIRT4 and SIRT1. Most importantly, SIRT4 negatively regulates SIRT1 expression via repressing glutamine metabolism. Besides, we identified H4K16ac and BRCA1 as new prime targets of SIRT4 in breast cancer.

**Conclusions:** These results demonstrate that SIRT4 exerts its tumor-suppressive activity via modulating SIRT1 expression in breast cancer and provide a novel cross-talk between mitochondrial and nuclear sirtuins.

## Introduction

Sirtuins (SIRTs) are nicotinamide adenine dinucleotide (NAD+)-dependent class-III histone deacetylases (HDACs) that belong to the silent information regulator 2 (SIR2) family. As the stress-responsive proteins that direct various post-translational modifications of histones and downstream non-histone targets, the seven mammalian sirtuins (SIRT1-7) are considered to be master regulators of several fundamental biological processes, including cell death and survival, chromatin remodeling, energy metabolism, aging, inflammation, and tumor development 1-3.

Among the sirtuin family, SIRT1 is the founding and the most extensively studied member. SIRT1 is ubiquitously expressed in the nucleus but shuttles between the nucleus and cytoplasm 4. Similar to other HDACs, aberrant SIRT1 expression is reported in multiple human malignancies, namely, gastric, colon, prostate, and ovarian cancers, as well as breast cancer 5-11. The implications of SIRT1 in breast cancer occurrence and progression have been investigated over recent years. However, the exact role is still controversial due to its contradictory functional roles.

Unlike SIRT1-3 and SIRT7, which are primarily lysine deacetylases, SIRT4 serves as both an ADP-ribosyl-transferase and lysine deacetylase. It is a relatively unstudied member of the SIRT family 12. SIRT4 is located within mitochondria and regulates catabolism of multiple nutrients 13. Despite the regulatory function in metabolism, SIRT4 was found to be involved in mediating cell cycle progression and signaling pathways like MEK/ERK or AMPK/mTORC 14-16. So far, emerging evidence on the role of SIRT4 as a tumor suppressor gene has been shown. According to The Cancer Genome Atlas (TCGA) database analysis, SIRT4 mRNA levels in human gastric, breast, bladder, colon, thyroid, and ovarian cancers were decreased compared with normal tissues 17, 18. Decreased SIRT4 protein levels were associated with poor prognosis in colon, lung, and esophageal cancer 16, 19, 20. Intriguingly, although more tumor tissue samples were found SIRT4 positive than adjacent nontumor tissue samples by immunohistochemical staining, low SIRT4 expression was associated with poor overall survival in breast cancers patients, especially in Luminal A patients 21. However, critical mechanisms linking reduced SIRT4 expression to breast cancer progression remain unclear. It has therefore been proposed that loss of BRCA1 function in basal stem cells results in tumor formation associated with a block in luminal differentiation 22.

In contrast, recent work showed an increase in luminal progenitor numbers in the breast tissue of BRCA1 mutation carriers and a correlation between the gene expression profile of normal human luminal progenitors and basal-like breast cancers. Compared with most sporadic breast cancers, those arising in carriers of BRCA1 mutations usually have distinctive pathological characteristics. A study suggests that a role for BRCA1 in the determination of stem-cell fate may explain this phenomenon 23.

This study is to identify a mechanism linking decreased SIRT4 expression to breast tumor-initiating cells (BTICs) regulation and cancer progression. Here, we show that SIRT4 possesses its tumor-suppressive effect on breast cancer by inhibiting glutamine metabolism and thereby affecting the protein levels of SIRT1, which modulates the acetylation patterns of histones H4 and regulates stemness via BRCA1.

## Materials and Methods

### Chemicals and Antibodies

Lipofectamine 2000 transfection and TRIZOL LS reagents were bought from Invitrogen (Grand Island, NY, USA). The DAB substrate kit has been purchased from Vector Laboratories, Inc (Burlingame, CA, USA). Abcam (Cambridge, MA, USA) provided antibodies towards SIRT4 (#: ab10140), SIRT1 (#: ab189494), H4K16ac (#: ab109463), H3K9ac (#: ab272105), Oct4 (#: ab19857), SOX2 (#: ab97959). Nanog (#: 8822), BRCA1 (#: 9010) and β-actin (#: 4970) antibodies were obtained from Cell Signaling Technology (Danvers, MA, USA). Unless, in any other case noted, all other used chemicals were from Sigma (St. Louis, MO, USA).

### Cell lines and cell culture

Normal mammary epithelial cell line MCF10A and breast cancer cell lines MCF-7, MDA-MB-231, BT549, MDA-MB-468 and HEK 293 Phoenix ampho cells were purchased respectively from the Cell Bank of different types culture collections of Shanghai Institute of Cell Biology and Chinese Academy of Sciences. As earlier mentioned, cell lines were regularly cultured. Typically, cell lines were kept at 37 °C in an environment comprising 5% CO_2_ in Dulbecco's modified Eagle medium or RPMI 1640 (Gibco) supplemented with 10% fetal bovine serum. Cells were passed every 1-2 days to keep logarithmic growth. All these cell lines were regularly screened for mycoplasma contamination by using the Universal mycoplasma detection kit, which was obtained from ATCC (Manassas, VA), and the last mycoplasma test was conducted in May 2017. Mycoplasma-free cell lines have been used in all our experiments.

### Patients and specimens

Randomly, samples were gathered from patients with breast cancer who underwent curative resection with informed consent between 2010 and 2015 at Qilu Hospital, Shandong University. All tissues were collected immediately after tumor resection, snap-frozen into liquid nitrogen, and then stored at -80 °C. Follow-up information was summarized at the end of December 2017 with an average observation interval of 75.4 months. Study protocols were endorsed by Shandong University's Hospital Ethics Committee. Based on the Helsinki declaration, written informed consent was obtained from patients.

### MTT Cytotoxicity assays

A total of 2,000 cells/well were plated 12 hours before treatment in 96 well plates. The cells were then handled for 72 hrs with specified agents. 10% v/v of 5 mg/ml 3-[4,5-dimethylthiazol-2-yl]-2,5-diphenyltetrazolium bromide (MTT) solution was introduced for a duration of 2 hr. The medium was then discarded, and the cells dissolved in DMSO (Sigma, St Louis, MO). Relative Cytotoxicity was determined by using a BMG Labtek plate reader to measure the absorbance at 570 nm. All expected experiments were conducted in triplicates, and the mean was calculated with SEM.

### Cell invasion and motility assay

Cell invasion was conducted in Matrigel (BD, Franklin Lakes, NJ, USA)-coated Transwell inserts (6.5 mm, Costar, Manassas, VA, USA) comprising 8-μm pores polycarbonate filters as described above. The inserts were covered with 50 μl of 1 mg/ml Matrigel matrix as it was recommended by the manufacturer. 2×10^5^ Cells in 200 μl of serum-free medium were plated in the upper chamber, whereas 600 μl of medium with 10% fetal bovine serum was added to the lower well. Cells that migrated to the lower surface of the membrane were fixed and stained after 24 hours of incubation. Five random fields were counted at about ×10 magnification for each membrane. The mean was calculated, and the information was displayed as mean ± s.d. from three independent triplicate tests. Motility assays were comparable to Matrigel's invasion assay except that the Transwell insert was not covered with Matrigel.

### HOECHST 33342 staining, Flow cytometry analysis, and sorting of SP

Cells were washed with PBS, detached from the culture plate with trypsin and EDTA, pelleted by centrifugation, and resuspended in 37 °C DMEM containing 2% FBS at 1×10^6^ cells/ml. Cell staining has been conducted as earlier outlined. Either the cells were incubated alone, or in combination with known ABC transporter inhibitor verapamil (50 μM, Sigma) at 5 μg/ml with Hoechst 33342 (Sigma, St, Louis, MO) for 90 min at 37 °C., The cells were centrifuged and resuspended after staining in HBSS (Invitrogen, Carlsbad, CA) containing 1μg/ml propidium iodide and maintained at 4 °C for flow cytometry evaluation and sorting. Cell evaluation and sorting were conducted on a 351- and 488-nm blue MoFlo cytometer (Dako Cytomation, Fort Collins, CO) fitted with a Coherent Enterprise II laser-emitting MLUV. The Hoechst 33342 emission was first divided using a short-pass 610-nm dichroic filter and the red and blue emissions were obtained through 670/30 and 450/65 nm bandpass filters, respectively.

### Quantitative RT-PCR

Total RNA extraction has been done by using Trizol reagent (Invitrogen), and cDNA was obtained by using SuperScript II Reverse Transcriptase purchased from Invitrogen. The ABI PRISM 7900HT sequence detection system performed the qRT-PCR, and it has been involved in data collection during the experiments.

### Immunoblotting

For immunoblotting, standard methods were used. Cells were lysed in the lysis buffer, and the Bradford method was precisely used to determine the total protein content. SDS-PAGE separated 30 μg of protein under optimal conditions and blotted to a polyvinylidene difluoride membrane (Millipore). Specific primary antibodies have been tested for membranes. Blots were washed and then blocked with respective secondary peroxidase-conjugated antibodies. Bands were visualized by chemiluminescence (Amersham Biosciences) as well.

### Confocal Immunofluorescence microscopy

By following the standard procedures, Cell lines were placed on culture slides (Costar, Manassas, VA, USA). After 24 hrs, the cells were rinsed with phosphate-buffered saline (PBS) and fixed with 4% paraformaldehyde in PBS. The cell membrane was permeabilized using 0.5% Triton X-100. These cells were then blocked for 30 min in 10 % BSA in PBS and then incubated with primary antibodies in 10 % BSA overnight at 4℃. After three washes in PBS, the slides were incubated for 1 hour in the dark with FITC-conjugated secondary goat anti-mouse or goat anti-rabbit antibodies (Invitrogen, Grand Island, NY, USA). After three consecutive washes, the slides were stained with DAPI for 5 min to visualize the nuclei and examined using a Carl Zeiss confocal imaging system (LSM 780) (Carl Zeiss, Jena, Germany).

### Clonogenic and sphere-formation assay

For clonogenic assays, we plated cells at 1,000 cells / well in Matrigel (MG) or methylcellulose (MC) at the proportion of 1:1 in 100-200 μl and counted colonies 1-2 weeks after placing. Sphere-formation culture has been conducted with slight changes. Single-cell suspensions were plated at a distinct density of feasible cells in 96-well plates of ultralow attachment (Costar, Manassas, VA, USA). Cells were cultivated in a serum-free mammary epithelial growth medium (MEGM), supplemented by 1:50 B27 (Invitrogen, Grand Island, NY, USA), 20 ng/ml epidermal growth factor (EGF), 20 ng/ml basic fibroblast growth factor (bFGF) (BD, Franklin Lakes, NJ, USA) and 10 μg/ml heparin (Sigma, St. Louis, MO, USA). Spheroid figures were counted after 7-10 days. Primary spheres were gathered for in-vitro propagation, dissociated into a single cell suspension and plated 96-well sheets in ultralow connection. The secondary spheroid figures were counted after 14 days of plating.

### Biopsies immunohistochemistry

Fixed tissues were sectioned at a size of 4 μm. Tissue pieces were stained according to the manufacturer's protocol with Biotin-Streptavidin HRP Detection Systems. Gently, sections were deparaffinized, and antigen repaired with sodium citrate and then incubated at room temperature with 3 % hydrogen peroxide for 10 minutes. Sections of the tissue were blocked with goat serum at 37 °C for 15 minutes and incubated at 4 °C overnight with the specified vaccine. Positive and negative staining regions were identified using Image-Pro Plus 5.1 software (Media Cybernetics, Silver Spring, MD, USA).

### *In vivo* tumorigenesis and metastasis assay

A group of 6 Balb/c nude mice was injected subcutaneously with infected cells into the left and right flanks for tumorigenesis assay. The tumor size was assessed using calipers to measure tumor dimensions for up to 42 days. Cells were resuspended in PBS for metastasis assay, and the cell suspension was injected into nude mice's tail veins. All animals were maintained under the guidelines of Shandong University and evaluated and approved by the Institutional Animal Care and Use Committee (Shandong University, Jinan, China). Food and water were supplied ad libitum.

### SIRT4 knockout mice

SIRT4 knockout** (**KO) mice were obtained from The Jackson Laboratory. All animals were numbered, and experiments were conducted in a blinded fashion. After data collection, genotypes were revealed, and animals assigned to groups for analysis. For treatment experiments, mice were randomized. None of the mice with the appropriate genotype were excluded from this study or used in any other experiments. Mice had not undergone prior treatment or procedures. All mice were fed a standard chow diet ad libitum and housed in pathogen-free facility with standard controlled temperature, humidity, and light-dark cycle (12 h) conditions with no more than five mice per cage under the supervision of veterinarians. All animal procedures were reviewed and approved by the Institutional Animal Care and Use Committee of Shandong University.

### Whole-mount staining

At the indicated ages, the fourth inguinal glands were dissected and spread on a glass slide. After 2 hours of acidic alcohol fixation, the tissues were hydrated and stained overnight in Carmine alum. Samples were then dehydrated, cleared, and mounted by xylene.

### Primary mouse mammary gland epithelial cell isolation

Preparation of the single-cell mammary gland was carried out as earlier outlined. Briefly, mammary thoracic and inguinal glands were dissected from mice, the tissues were digested at 37 °C for 6-8 h in DMEM/F12 supplemented with 10% FBS and 1% P/S and 750 U/ml Collagenase and 250 U/ml hyaluronidase. After this step, the organoids were gathered by centrifugation then treated individually with trypsin (0.5%) and dispase (5 mg/ml); Ammonium chloride was used for red blood cell lysis after centrifugation. Unless otherwise mentioned, all reagents were acquired from stem cell technology.

### Mouse mammary stem cell analysis

Mammary glands were dissected from mice aged seven weeks. Mammary stem cells were evaluated after mechanical dissociation. For isolation of stem/progenitor cells, the following antibodies were used: CD49f and CD24 (Stem cell technology, eBioscience). Blocking was done for 10 min with rat serum. Cells were stained for 30 min on ice and washed with staining media. Then, cells were resuspended in staining media containing 7-aminoactinomycin D (1 μg/ml) to stain dead cells. Cells were analyzed using an LSR II, Flow-jo, and sorted Mo flow cell sorter.

### Establishment of SIRT4 stable expression and SIRT4, SIRT1 cell lines knockdown

PBabe.puro retroviral construct containing human SIRT4 cDNA and pSuper.retro.puro with human SIRT4 shRNA was prepared as previously described 15. The generation of retrovirus supernatants and the transfection of breast cancer cells were carried out. Infected cells were chosen by adding 2 μg/ml puromycin to the 48-hour culture medium and then retained in a complete medium with 0.5 μg/ml puromycin. The above protocols also generated empty retroviral-infected stable cell lines. shRNA against SIRT1 expressed in the pSuper vector was prepared as earlier outlined. The generation of retrovirus supernatants and the transfection of breast cancer cells were chosen as outlined above, except for infected cells by adding 400 μg/ml of G418. The expression of SIRT4 and SIRT1 was verified by the study of qRT-PCR and Western blotting.

### Measurement of glutamine and glucose

The BioProfile FLEX analyzer (Nova Biomedical) was used to measure glutamine, ammonia, glucose, and lactate concentrations in culture media. Briefly, new media were introduced to a 6-well cell plate, and media metabolite concentrations were analyzed 6-9 hrs later and normalized to the number of cells in each well.

### Chromatin immunoprecipitation and sequencing

ChIP protocol was adapted from the MagnaChIP kit protocol (Millipore). Cells were fixed with 1% formaldehyde, lysed with Cell Lysis, and then Nuclear Lysis buffers respecting concentration of 2×10^7^ cells per mL and finally sonicated (30-min cycle on Covaris apparatus; KBioscience). Sheared chromatin was immunoprecipitated overnight using the following antibodies: anti-H4K16ac, anti-H3K9ac, and rabbit IgG. 1/10 of the sheared chromatin was used as a reference (Input). The immune complex collection was realized with Protein G Sepharose (Sigma-Aldrich, P3296), 1h30 at +4°C. For GFP-ChIP, GFP-Trap agarose beads were used (Chromotek). Rinses were done according to the MagnaChIP kit protocol with low salt, high salt, and LiCL immune complex wash buffers. Finally, elution was performed according to the IPure Kit protocol (Diagenode, Cat No C03010012) following the manufacturer's instructions. Enriched DNA from ChIP and Input DNA fragments were end-repaired, extended with an 'A' base on the 3′end, ligated with indexed paired-end adaptors (NEXTflex, Bioo Scientific) using the Bravo Platform (Agilent), size- selected after 4 cycles of PCR with AMPure XP beads (Beckman Coulter) and amplified by PCR for 10 cycles more. Fifty-cycle single-end sequencings were performed using Illumina HiSeq 2000 (Illumina, San Diego, CA). Reads were aligned to human genome hg19 with BWA aln (v0.7.5a) and peak calling assessed using MACS 2.0 with a q-value cut-off. Peak-calling analyses for each ChIP-seq were realized independently. Then we created one specific list of peaks for each mark, including familiar peaks in at least 2 replicates. Annotation and motif analyses have been done with HOMER (v4.7.2), with a p-value <10^-30^. Integrative Genomics Viewer (IGV 2.3.34) was used for representation. Intensity profiles of ChIPseq data were done using ngsplot version 2.61.

### Chromatin Immunoprecipitation-qPCR Analysis

For ChIP analysis, 3.0 ml of purified chromatin was used with a Sybr green master mix (Applied Biosystems, Foster City, CA, USA), and qPCR were analyzed using ABI 7900 thermocycler. Each response was conducted in triplicate, and the tests were repeated at least twice in order to verify reproducibility. Values for the threshold cycle (Ct) were acquired for each gene or genomic region, and information was analyzed using the standard curve technique. For ChIP-qPCR assessment, values were standardized to the input control. They expressed as a fold rise over-enrichment identified using IgG. The median expression ± S.E.M. has been recorded.

### Luciferase Reporter Assays

Cells were transduced by BRCA1 reporter (Signal BRCA1 Lentivirus Reporter, QIAGEN) and chosen for Puro Resistance. The reporter activity was evaluated using the Dual-Glo Luciferase Assay System (Promega) and normalized to EF1 α-renilla luciferase.

### Microarray analysis

The microarray studies were conducted by Shanghai Biotechnology Corporation (Shanghai, China). Total RNA was separated from three replicate specimens of human podocytes using TRIzol reagent (Invitrogen). Agilent Whole Human Genome Oligo Microarray (Agilent Technologies, Santa Clara, CA, USA) was used for transcriptome analysis. Microarray information was standardized using GeneSpring GX software (Agilent Technologies), and genes were classified into numerous pathways using the David database accessible on (https:/david.ncifcrf.gov/).

### Mass spectrometric metabolomics analysis

Mouse mammary gland specimens were washed overnight in a speed vac and then homogenized in PBS (pH 7.4) using an Omni International bead homogenizer device at 6.45 m/s for 30 sec. Homogenate was obtained using CHCl3: MeOH (2:1) to separate polar and nonpolar metabolites. Samples were centrifuged at 3000 rpm for 5 min and then analyzed with LC-MS/MS using the selected reaction monitoring (SRM) method with positive/negative ion polarity switching to a Xevo TQ-S mass spectrometer. The peak areas integrated using MassLynx 4.1 (Waters Inc.) have been normalized to the respective protein concentrations, and the resulting peak areas have been subjected to relative quantification analysis using Metaboanalyst 3.0 (www.metaboanalyst.ca).

### Analysis of TCGA breast cancer data

By using the co-occurrence tool at cBioPortal for Cancer Genomics, a TCGA breast cancer data set was used to correlate the expression of SIRT4 and SIRT1 with an expression of 286 ribosomal processing genes. P-values were obtained from Fisher's precise T-test. The TCGA LADC dataset was also considered for an association of SIRT4 mRNA with disease-free and overall survival was calculated using the cBioPortal survival analysis tool and the log-rank test to evaluate statistical significance.

### GSEA analysis

Global mRNA expression profiles of breast cancer samples from Gene Expression Omnibus (GEO) were subject to Gene Set Enrichment Analysis (GSEA) using GSEA 2.0.9 software available on (http:/www.broadinstitute.org/gsea/) to reveal the connection between SIRT4 expression and stem cell pathway signature.

### Statistical analysis

The TCGA BC information was used to evaluate SIRT4 genomic copy modifications. TCGA information analysis was performed using the Xena method of USDSC, which is available on (http://xena.ucsc.edu). Statistical analysis was conducted using the statistical software program called SPSS (IBM. Armonk, New York, USA. Student two-tailed t testing was also used to determine comparisons between distinct groups). Results are presented as mean ±SD; probabilities less than 5% (*p* < 0.05) were considered to be statistically significant.

### Ethics Committee Approval and Patient Consent

This study was reviewed and approved by the Ethics Committee of the Qilu Hospital of Shandong University and the Second Hospital of Shandong University.

## Results

### SIRT4 expression is downregulated in breast cancer and related to mammary gland development and stemness

**Figure 1:**
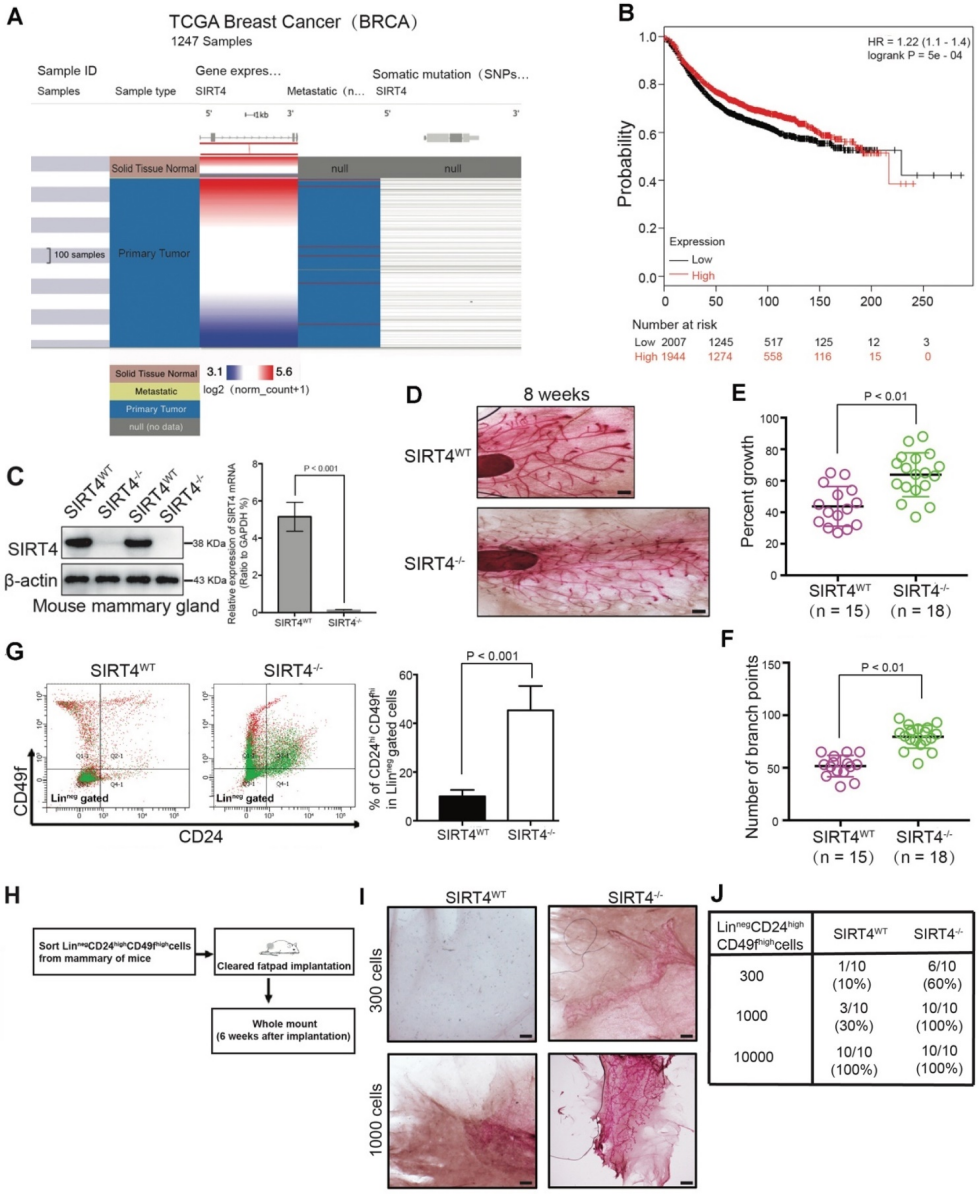
** SIRT4 expression is downregulated in breast cancer and related to mammary gland development and stemness.** (**A**) SIRT4 expression levels in breast cancers compared to healthy tissues from the TCGA data set of 1247 samples. (**B**) Kaplan-Meier analysis indicating the overall survival of breast cancer patients with high (red) (n = 1944) or low (black) (n = 2007) SIRT4 expression. (**C**) SIRT4 KO mice were obtained from The Jackson Laboratory. SIRT4 knockout efficiency was measured by immunoblotting (left panel) and qRT- PCR (right panel). (**D**) Whole-mount analyses were conducted on 8-week-old SIRT4 wild-type (SIRT4^WT^) (n = 15), and SIRT4^-/-^ (n = 18) mice and representative images of mammary gland side-branches are shown. (**E**) Percent of growth. (**F**) The number of mammary gland side-branches was quantified. (**G**) Distribution of Linneg mouse mammary cells according to their expression of CD24 and CD49f was analyzed on 8-week-old SIRT4^-/-^ mice and littermate controls (left). Mammary stem cells (MSCs), according to their expression of CD24hiCD49fhi in Linneg (right), were quantified by a flow cytometric analysis. (**H, I and J**) Schematic representation of limiting dilution transplantation experiments with LinnegCD24hiCD49fhi MSCs (H). A total of 300, 1000, or 1 x 10^4^ LinnegCD24hiCD49fhi MSCs isolated from 8-week-old SIRT4^-/-^ mice and littermate controls were injected into the cleared fat pad of 3-week-old FVB/NJ female mice. Whole-mount analyses were then conducted at 6 weeks after injection (I, J). Representative images of mammary gland side-branches are shown (I). The resulting data were analyzed by the Chi-square test (p < 0.001) (J). Scale bars, 100 µm (D) and 50 µm (I).

Consistent with other studies 17, 24, our analysis performed based on data from the TCGA database validated that *SIRT4* was downregulated in breast cancer (Figure 1A; Figure S1A-C). Similarly, the analysis of TCGA and 3 Gene Expression Omnibus (GEO) datasets (GSE2034, 25307, 7390) showed that decreased *SIRT4* expression correlated with increased risk of disease progression and poor clinical outcome (Figure 1B; Figure S1D). Besides, immunoblotting and immunohistochemistry staining also showed a significant decrease in SIRT4 protein levels in breast tumor tissues (Figure S2).

To assess the role of SIRT4 in mammary epithelium, whole-mount analyses were conducted on 8-week-old *SIRT4* wild-type (*SIRT4^WT^*) (n = 15) and *SIRT4^-/-^* (n = 18) mice (Figure 1C). Noticeably, *SIRT4^-/-^* mice showed an increased ductal side-branching (p < 0.01, Figure 1D-F) and higher numbers of mammary stem cells (MSCs; defined as CD24^hi^CD49f^hi^ in Lin^neg^ gated cells) (p < 0.001, Figure 1G). Meanwhile, we found that the deletion of SIRT4 contributed to terminal end bud (TEB) development in the intact mammary glands (Figure S3A). Importantly, limiting dilution transplantation experiments with Lin^neg^CD24^hi^CD49f^hi^ MSCs demonstrated that the increase in lobuloalveolar structures in *SIRT4^-/-^* mice directly relates to MSCs (Figure 1H-J). Together, these observations indicate that SIRT4 probably disrupts mammary gland development and that the deletion of SIRT4 strikingly promotes TEB development that grows out from MSCs.

### SIRT4 deletion promotes mammary tumorigenesis

**Figure 2:**
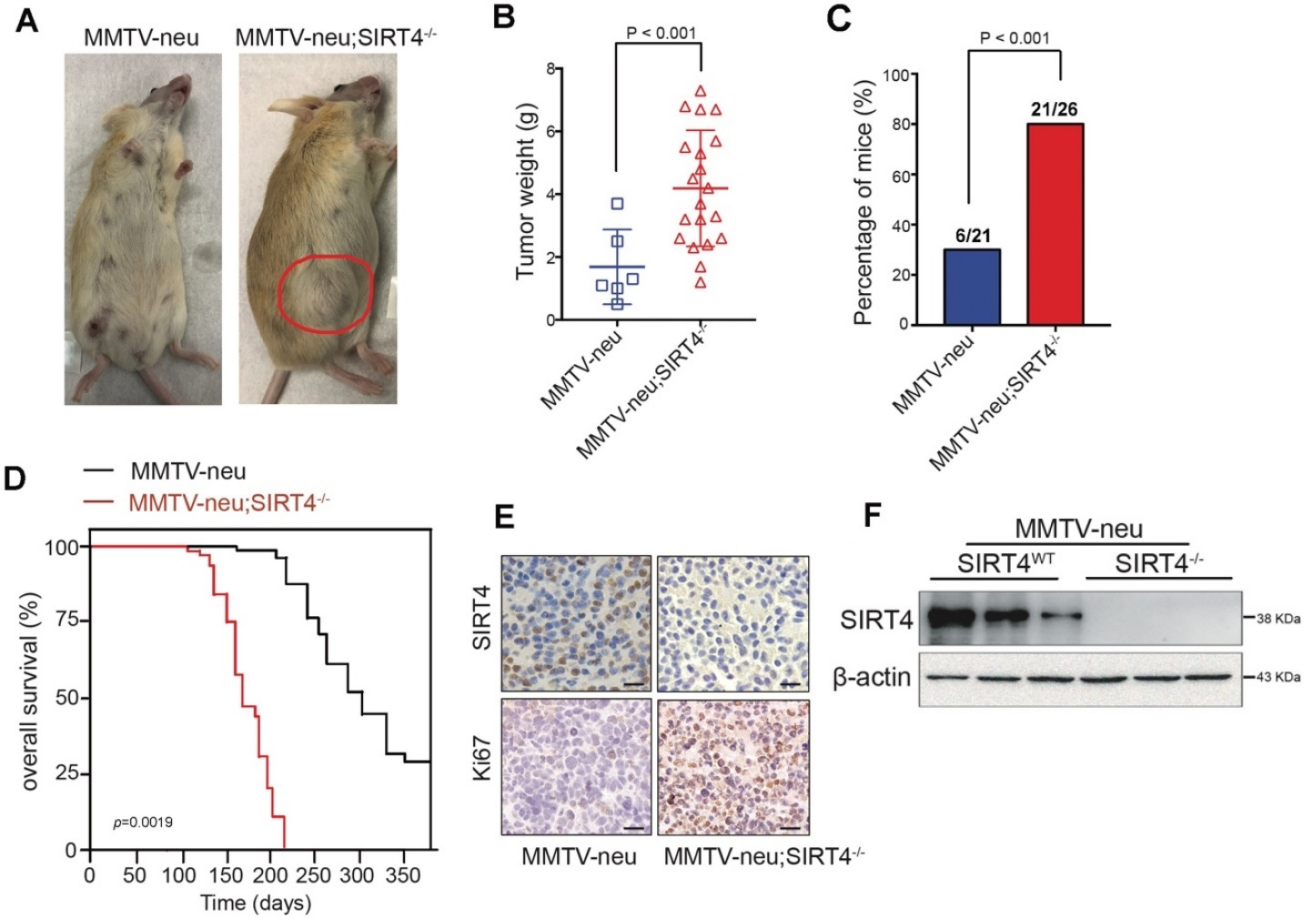
** SIRT4 deletion promotes mammary tumorigenesis.** (**A**) SIRT4^-/-^ mice were crossed with MMTV-Neu mice to generate MMTV-Neu transgenic mice in wild-type and SIRT4-null genetic backgrounds. The mammary tumor was developed at an average age of 240 days. Representative images are shown. Using these mice, we monitored (**B**) tumor weight, (**C**) tumor incidence, (**D**) overall survival time, (**E**) Ki67 and SIRT4 stained tumor sections, (**F**) SIRT4 protein expression in tumors isolated from MMTV-neu and MMTV-neu; SIRT4^-/-^ mice. Data are means ± SEM. *p* < 0.01; *t*-test (B and C). Scale bars, 50 µm (E).

To evaluate the relative contribution of SIRT4 in mammary tumorigenesis, we then crossed *SIRT4*^-/-^ mice with MMTV-Neu mice, which mimic the human luminal phenotype and develop spontaneous mammary tumors after acquiring either additional mutations or epigenetic modifications 25, 26 Figure 2F and Figure S4A showed the SIRT4 expression in crossed mice. The tumors developed in *MMTV-neu*;* SIRT4*^-/-^ mice showed high tumor formation and lung metastasis compared to control models (Figure 2A-C; Figure S4D). The overall survival rate of *MMTV-neu*;* SIRT4*^-/-^ mice was significantly low compared with MMTV-Neu mice (Figure 2D). Besides, SIRT4 knockout significantly increased cell proliferation as measured by Ki67 expression and MSC population (Figure 2E; Figure S4B-C). Together, these results suggest that SIRT4 plays a key role in mammary tumor development, which might implicate MSC formation.

### SIRT4 inhibits self-renewal and expansion of breast tumor-initiating cells (BTICs)

**Figure 3:**
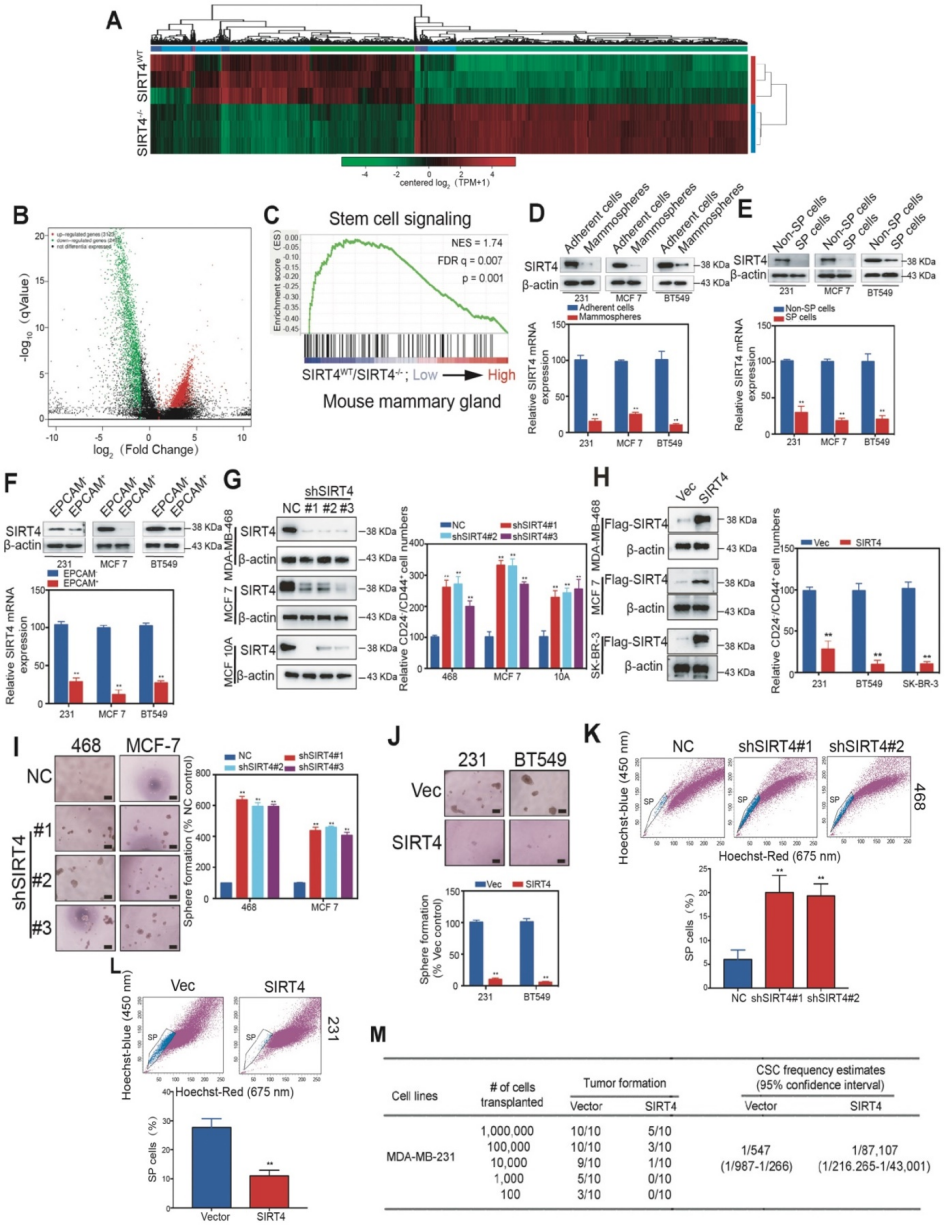
** SIRT4 inhibits self-renewal and expansion of breast tumor-initiating cells (BTICs).** (**A**) Heatmap summarizing genes differentially expressed in SIRT4^-/-^ compared to SIRT4^WT^ mice. (**B**) Volcano plot displaying differentially expressed genes. Up-regulated genes (3123) are highlighted in red. Down-regulated genes (2477) are highlighted in green. Black dots represent genes not differentially expressed. (**C**) Enrichment of a stem cell signaling in GSEA analysis of genes altered as described above. (**D, E, F**) Immunoblotting (upper panel) and mRNA expression (bottom panel) of SIRT4 in CSC-enriched mammospheres (D), side population (SP) cells (E), and EPCAM+ cells (F) as well as their corresponding controls, i.e., adherent cells (A), non-SP cells (C), and EPCAM- cells (E). (**G**) Quantification of CD44^+^/CD24^-^ subpopulations (right panel) and immunoblotting of SIRT4 expression (left panel) in MDA-MB-231, MCF-7, and BT549 cells transfected with sh-SIRT4 or negative control (NC). (**H**) Quantification of CD44^+^/CD24^-^ subpopulations (right panel) and immunoblotting of SIRT4 expression (left panel) in MDA-MB-468, MCF-10A, and SK-BR-3 cells transfected with SIRT4 or control vector (Vec). (**I, J**) Sphere formation efficiency of cells described in G (I) and H (J), respectively. (**K, L**) Hoechst SP assay of cells described in G (K) and H (L), respectively. (**M**) Tumor formation ability of MDA-MB-231 cells expressing control (Vector) or SIRT4 vector. The transfected MDA-MB-231 cells were assayed for the ability to form tumors by subaxillary injection of 1 × 10^6^, 1 × 10^5^, 10,000, 1,000, and 100 cells into nude mice. The numbers of tumors formed and the number of injections that were performed is listed for each population. Data are means ±SEM. ^**^p < 0.01; *t*-test. Scale bars, 100 µm (I and J).

To gain insight into the mechanism by which SIRT4 regulates mammary tumorigenesis, we performed RNA-seq with mammary cells from *SIRT4^WT^* and *SIRT4^-/-^* mice (Figure 3A). A total of 3123 genes were found to be up-regulated, while 2477 genes were negatively correlated with SIRT4 depletion (Figure 3B). The top 50 genes significantly differentially expressed were summarized in the heatmap (Figure S5). Gene Ontology (GO)-analysis revealed the enriched signatures related to endogenous SIRT4-dependent transcription in breast cancer, namely, stem cell signaling (Figure 3C). Consistently, the reduced SIRT4 expression was observed in the CSC-enriched populations, including mammospheres, side population (SP) cells, and EPCAM+ cells (Figure 3D-F).

To further address the role of SIRT4 in cancer stemness, we next generated MDA-MB-231, BT549, and SK-BR-3 cells overexpressing SIRT4, and MDA-MB-468, MCF-7 as well as MCF-10A cells knocking down endogenous SIRT4 via lentiviral shRNA (Figure 3G-H; Figure S6; Figure S7A-D). As expected, the ablation of SIRT4 caused an evident increase of CD24^-^CD44^+^ populations, sphere formations, and SP cells, while forced expression of SIRT4 led to a noticeable reduction (Figure 3I-L). In order to test whether ectopic expression of SIRT4 could succeed in altering the tumor-initiating frequency, we injected transformed MDA-MB-231 cells with serial dilutions into nude mice. None of the mice that were injected with 10^3^ SIRT4-expressing MDA-MB-231 cells formed tumors (0 of 10 injected hosts), whereas 5 tumors arose when 10^3^ cells expressing a control vector were injected (Figure 3M). Thus, excessive expression of SIRT4 considerably decreased the number of breast tumor-initiating cells.

Meanwhile, MTT, colony formation, and bioluminescence imaging assay were performed to measure the oncogenic phenotypes of SIRT4-expressing MDA-MB-231 as well as SIRT4-deficient MCF-7 cells *in vitro* and *in vivo* (Figure S7E-J). In line with previous evidence, SIRT4 overexpression disrupted the proliferation, colony formation, and xenograft tumor formation of MDA-MB-231 cells, while ablation of SIRT4 caused the opposed effects on MCF-7 cells. Similarly, SIRT4 overexpression and deficiency lead to a robust downregulation or up-regulation of migration, mesenchymal markers, and liver as well as lung metastasis, respectively (Figure S8). The findings mentioned above show that SIRT4 plays a crucial role in the regulation of BTIC self-renewal and expansion and might protect mice from mammary tumorigenesis and lung metastasis by blocking MSC formation.

### Proteome-wide analysis of SIRT4 deficiency-induced expression in mouse mammary gland cells

**Figure 4:**
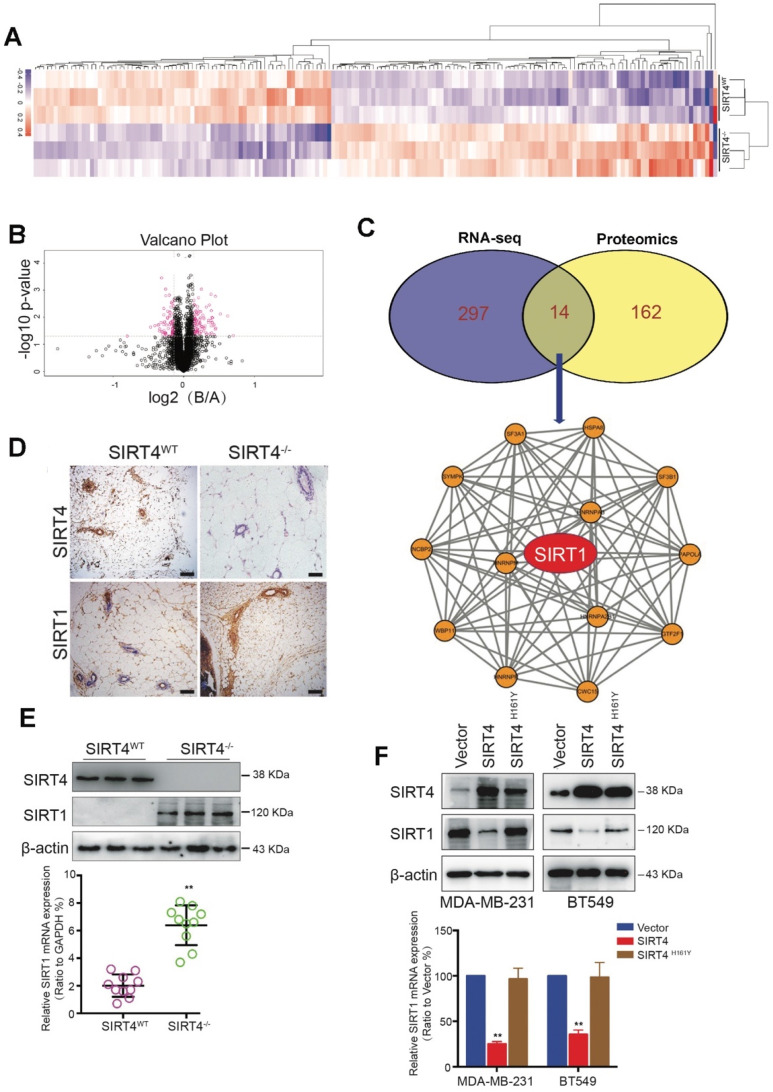
** Proteome-wide analysis of SIRT4 deficiency-induced expression in mouse mammary gland cells.** (**A**) Heatmap depicting proteins differentially expressed in mammary epithelial cells from SIRT4^WT^ and SIRT4^-/-^ mice. Up-regulated proteins are highlighted in pink. Down-regulated proteins are highlighted in purple. (**B**) Volcano plot displaying differentially expressed proteins in SIRT4^-/-^ compared to SIRT4^WT^ mice. (**C**) Venn diagram showing the overlap of genes (purple) and proteins (yellow) differentially expressed in mammary epithelial cells from SIRT4^WT^ and SIRT4^-/-^ mice (upper); Protein-Protein-Interaction Network including the 14 overlapped proteins in Venn diagram (bottom). (**D**) Representative IHC staining images of SIRT4 and SIRT1 in tumor sections isolated from SIRT4^WT^ and SIRT4^-/-^ mice. (**E, F**) Immunoblotting (upper panel) and mRNA expression (bottom panel) of SIRT1 in tumors isolated from SIRT4^-/-^ and SIRT4^WT^ mice (E), and in MDA-MB-231 and BT549 cells transfected with control (Vector), SIRT4 or mutated SIRT4 (H161Y) vector (F). Data are means ±SEM. ^**^p < 0.01; *t*-test (E and F). Scale bars, 100 µm (D).

To further identify the potential downstream targets of SIRT4, we performed the proteome-wide analysis with mammary epithelial cells from *SIRT4^WT^* and *SIRT4^-/-^* mice (Figure 4A-B). As a result, a total of 176 proteins was significantly altered by SIRT4 ablation. Among these proteins, 14 proteins were overlapped with the genes identified by RNA-seq (Figure 4C top). Subsequent functional network analysis revealed the core position of SIRT1 (Figure 4C bottom), which was up-regulated by SIRT4 deficiency. Since SIRT1 has been reported to be involved in tumor initiation and progression as well as cancer stemness, we hypothesized that SIRT1 might be a potential target of SIRT4.

In agreement with the hypothesis, SIRT4 deficiency increased SIRT1 expression at both mRNA and protein levels (Figure 4D-E; Figure S9). In comparison, overexpression of SIRT4 caused reduced SIRT1 expression, which was not observed in MDA-MB-231 and BT549 cells overexpressing catalytically inactive SIRT4 H161Y mutation (Figure 4F). Consistently, the reverse relationship between SIRT4 and SIRT1 proteins was further verified in human mammary tissues by IHC staining (Figure S10). The above findings show that SIRT4 negatively regulates SIRT1 expression in breast tumors.

### SIRT4 deficiency down-regulates BRCA1 expression

**Figure 5:**
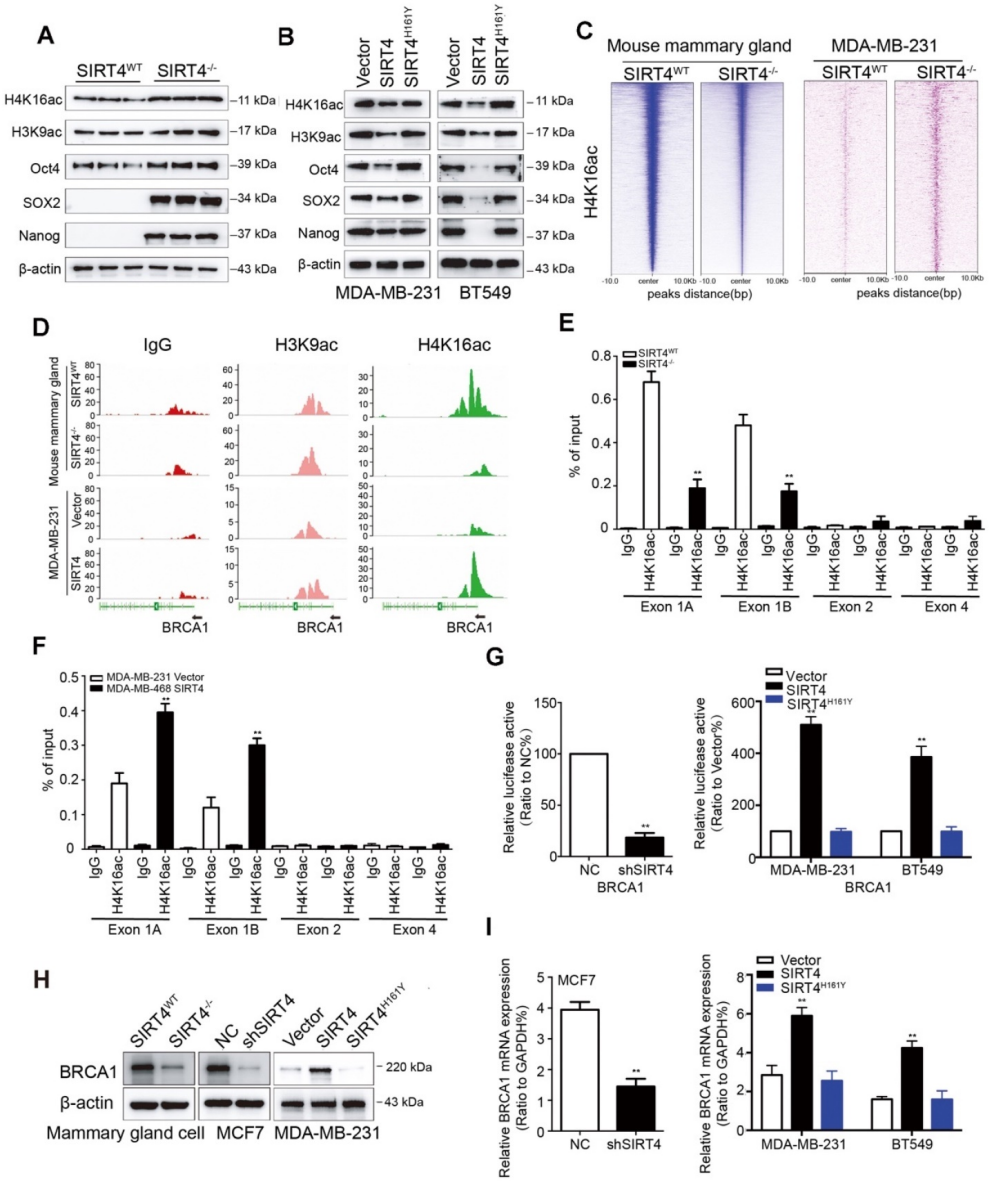
** SIRT4 deficiency down-regulates BRCA1 expression.** (**A, B**) Immunoblotting of acetyl-histone H4 at lys16 (H4K16ac), acetyl-histone H3 at lys9 (H3K9ac), Oct4, SOX2, and Nanog in mammary cells isolated from SIRT4^-/-^ as well as SIRT4^WT^ mice (A), and in MDA-MB-231 (left panel) as well as BT549 cells (right panel) transfected with control (Vector), SIRT4 or mutated SIRT4 (H161Y) vector (B). (**C**) Heatmap summarizing chromatin immunoprecipitation (ChIP)-seq data for H4K16ac, comparing mammary cells from SIRT4^WT^ and SIRT4^-/-^ mice (left panel), as well as MDA-MB-231 cells transfected with control (Vector) or SIRT4 vector (right panel). Profiles are centered on H4K16ac binding peaks and depict signal intensity (relative fold enrichment) with green color. (**D**) ChIP signal of IgG (left panel, red peaks), H3K9ac (middle, blue peaks), and H4K16ac (right panel, green peaks) in the indicated genomic regions of BRCA1 in cells described in C. (**E, F**) Mammary cells from SIRT4^WT^ as well as SIRT4^-/-^ mice (E) and transformed MDA-MB-468 cells (F) as described in C and Fig.3G were chromatin immunoprecipitated for IgG and H4K16ac. Pull-down at the putative H4K16ac binding sites was assessed by qRT- PCR and calculated as the percentage of IgG input. Error bars are SEM for 3 technical replicates. (**G**) Luciferase reporter assays showing the impact of SIRT4 deletion (left), as well as overexpression of SIRT4 and its mutation (H161Y) on BRCA1 promoters (right panel) in SIRT4^-/-^ as well as SIRT4^WT^ mammary cells, and MDA-MB-231 as well as BT549 cells, respectively. (**H, I**) Immunoblotting (H) and mRNA expression (I) of BRCA1 in cells described above. Data are means ±SEM. ^**^p < 0.01; *t*-test.

SIRT1 has been reported to modulate the acetylation patterns of histones H3 and H4 and regulate both stemness and metastasis in breast cancer 27, 28. Considering the inverse relationship between SIRT4 and SIRT1, we next assessed the effect of SIRT4 on acetyl-histone H3 at lys9 (H3K9ac) and acetyl-histone H4 at lys16 (H4K16ac) as well as stem cell markers like Oct4, Sox2, and Nanog by immunoblotting. As expected, SIRT4 deficiency increased the expression of these proteins. At the same time, overexpression caused a reduction in the expression of these proteins, and this was not observed in MDA-MB-231 and BT549 cells overexpressing catalytically inactive SIRT4 H161Y mutation (Figure 5A-B; Figure S11A).

Next, we assessed genome-wide H4K16ac binding by chromatin immunoprecipitation/high-throughput sequencing (ChIP-seq) analysis in mammary cells from *SIRT4^WT^* and *SIRT4^-/-^* mice and transformed MDA-MB-231 cells overexpressing SIRT4 or control vectors, respectively. As expected, the analysis of enriched loci (peaks) in *SIRT4^-/-^* and transformed MDA-MB-231 cells indicated that overall H4K16ac signals were lower and exhibited a short peak on the promoter region of *BRCA1* in mammary cells from* SIRT4^-/-^* mice and MDA-MB-231 cells transfected with control vectors (Figure 5C-D), suggesting histone acetylation is associated with activation of this promoter. To figure out whether this is the case, mammary cells from* SIRT4^WT^* and* SIRT4^-/-^* mice, together with transformed MDA-MB-231, MCF-7, MDA-MB-468, and BT549 cells as described above were chromatin immunoprecipitated (ChIP) for IgG and H4K16ac, followed by qPCR, which validated the ChIP-seq result and demonstrated low enrichment for H4K16ac -bound sequences at Exon 1 rather than Exon 2 and 4 (Figure 5E**-**F; Figure S11B-D). Consistently, luciferase reporter assays in transformed MDA-MB-231 and BT549 cells showed that ectopic expression of SIRT4 activated the promoter of* BRCA1*, which was fully rescued by the catalytically inactive mutant of SIRT4 (H161Y) (Figure 5G), suggesting an essential role of its catalytical activity in regulating *BRCA1* transcription. Similarly, SIRT4 deficiency downregulated *BRCA1* at both mRNA and protein levels, whereas SIRT4 overexpression caused the opposite effect (Figure 5H-I; Figure S12A-B). Additionally, the positive relationship between SIRT4 and BRCA1 expression was further verified by data from 3 GEO datasets (GSE1456 and 2034) and in human mammary tissues by IHC staining (Figure S12C-D). These data together provide compelling evidence that SIRT4 promotes histone acetylation at the promoter region of *BRCA1* to promote its transcription.

### SIRT1 is required for SIRT4 deficiency-induced BCSC phenotype

**Figure 6:**
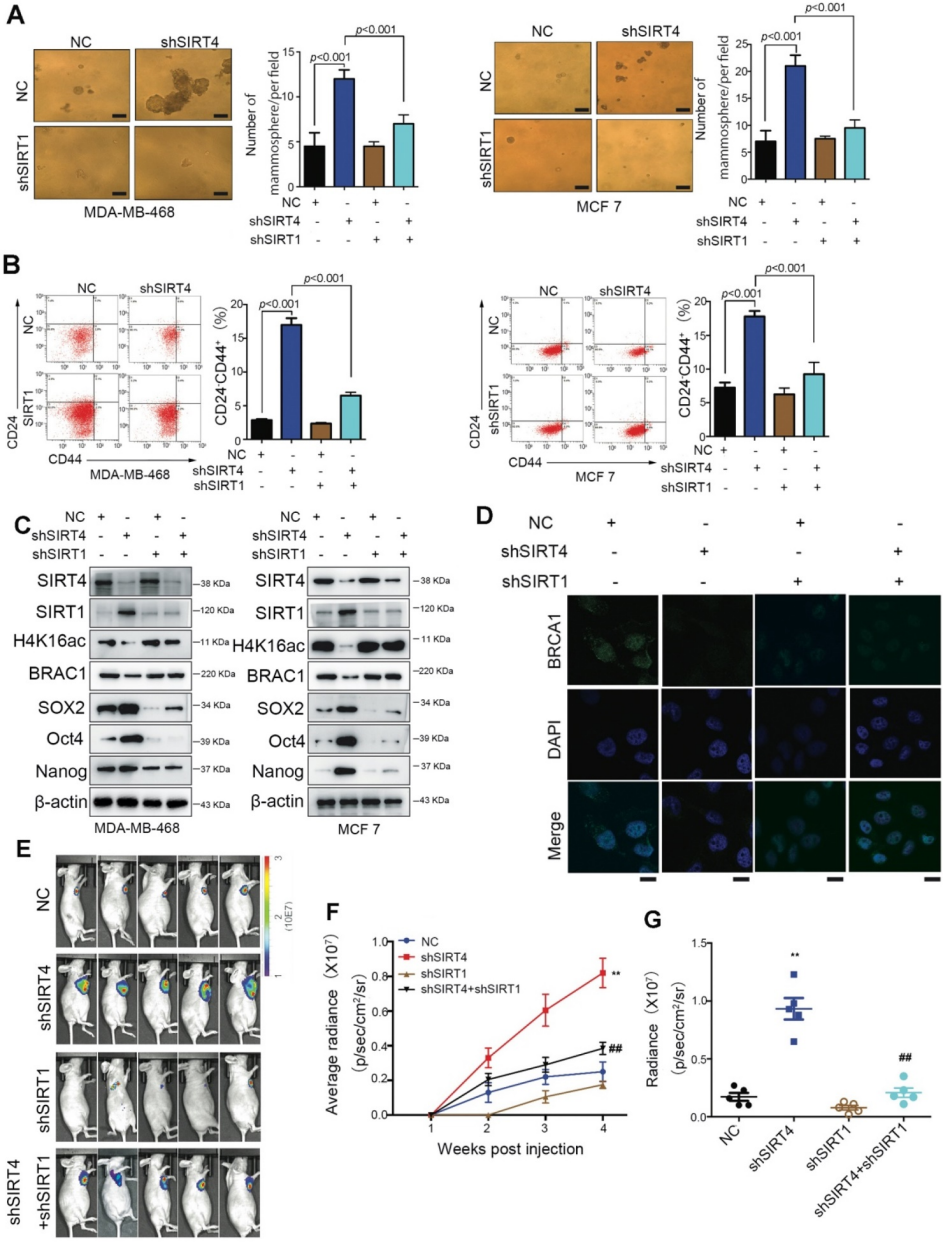
** SIRT1 is required for the SIRT4 deficiency-induced BCSC phenotype.** (**A**) Sphere formation efficiency of MDA-MB-468 (left) and MCF-7 cells (right panel) transfected with different combinations of control vector, sh-SIRT4, and sh-SIRT1. (**B**) Quantification of CD44^+^/CD24^-^ subpopulations (**C**) SIRT4, SIRT1, H4K16ac, BRCA1, SOX2, Oct4, and Nanog in MDA-MB-468 (left) and MCF-7 cells (right panel) described above. (**D**) Immunofluorescence images of cells described in (A) stained with antibodies against BRCA1/DAPI. (**E**) Representative ventral view images of bioluminescence from mice described above. (**F**) Tumor volume. (**G**) Quantification of E. Data are means ±SEM. ^**^p < 0.01; *t*-test. Scale bars, 100 µm (A) and 20 µm (D).

To prove the involvement of SIRT1 in SIRT4-deficiency-induced malignancy, we established stable breast cancer cell lines (MDA-MB-468 and MCF-7) expressing two independent shRNAs that target SIRT1 and SIRT4. As anticipated, downregulation of 15 CD24^-^CD44^+^ populations were observed in both cell lines (Figure 6A-B). Besides, the evaluation of stem cell markers further demonstrated that the stemness phenotype favored by SIRT4-deficiency was revoked by SIRT1 loss as well. Coincident observations were achieved by immunoblotting and immunofluorescence that SIRT1 deficiency completely abrogated the alterations of SIRT4 loss on the potential downstream targets, H4K16ac and BRCA1 (Figure 6C-D; Figure S13A-B). *In vivo* evidence from mouse xenografts injected with the double knockdown cells revealed that the ablation of SIRT1 expression counteracted the effects of SIRT4 decline on tumor growth (Figure 6E-G), which is consistent with the IHC data of Ki67 staining (Figure S13C). These data altogether indicate that repression of SIRT1 by SIRT4 is vital for blocking the CSC phenotype and tumor progression in breast cancer.

### Glutamine metabolism disorder mediates SIRT4-induced SIRT1 inhibition in breast cancer cell

**Figure 7:**
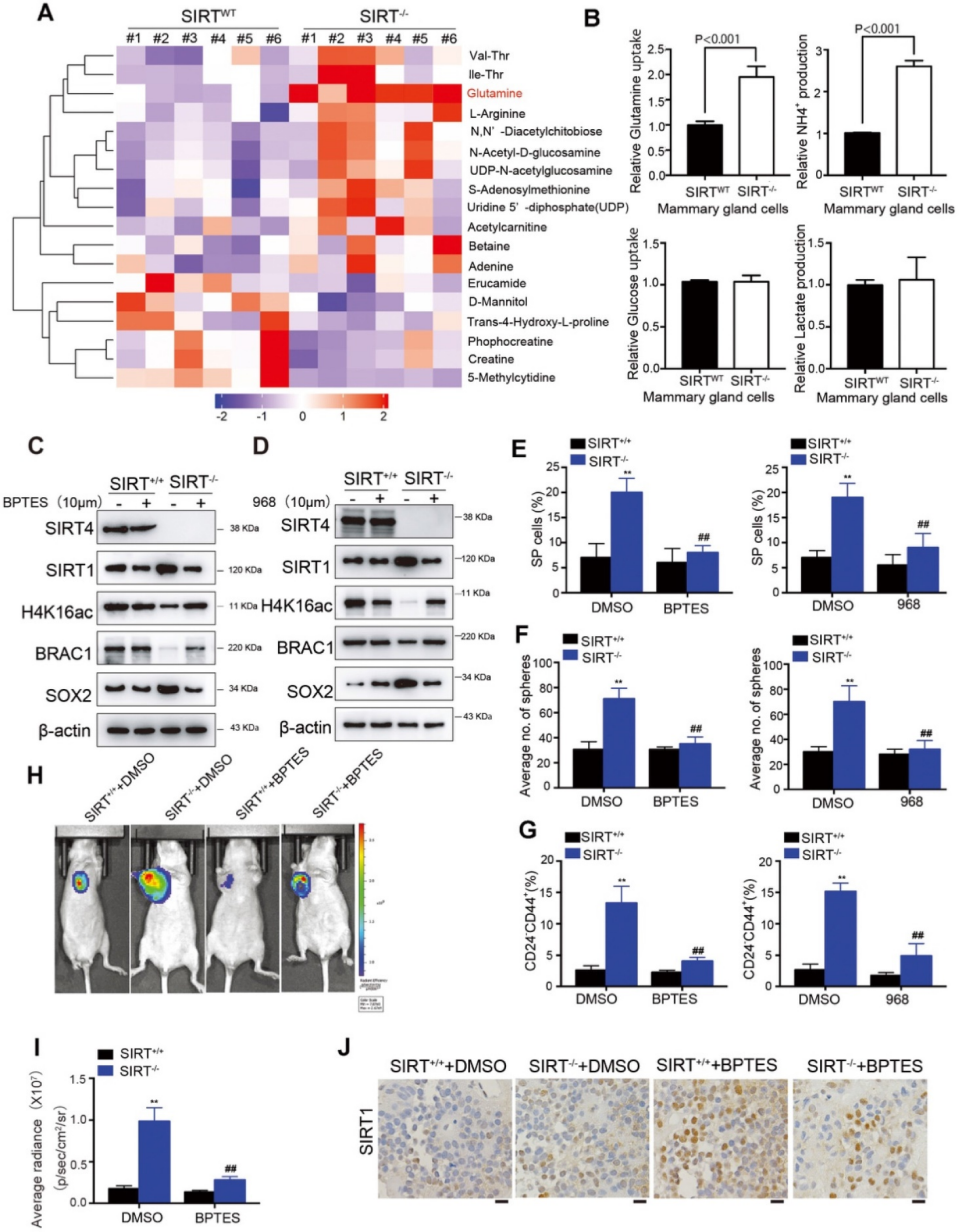
** Glutamine metabolism disorder mediates SIRT4-induced SIRT1 inhibition in breast cancer cells.** (**A**) Heatmap showing the changes in metabolites levels between SIRT4^WT^ and SIRT4^-/-^ mice. Up-regulated metabolites are highlighted in red. Down-regulated metabolites are highlighted in purple. (**B**) Measurement of Glutamine uptake (upper left), NH4+ production (upper right), Glucose uptake (bottom left), and lactate production (bottom right panel) in mammary epithelial cells from SIRT4^WT^ and SIRT4^-/-^ mice. (**C, D**) Immunoblotting of indicated proteins isolated from SIRT4^WT^ and SIRT4^-/-^mammary cells with or without BPTES (10 µM) (C) and 968 (10 µM) (D) treatment. (E, F, G) Quantification of Hoechst SP assay (**E**), sphere formation efficiency (**F**), and CD44+/CD24- subpopulations (**G**) in SIRT4^WT^ and SIRT4^-/-^mammary cells with or without BPTES (left) and 968 (right panel) treatment. (**H, I**) Representative ventral view images of bioluminescence from mice with injections of cells described above (H) and its quantification (I). (**J**) Representative IHC staining images of BRCA1 in tumor sections isolated from mice described in H. Data are means ± SEM. ^**^p < 0.01; *t*-test. Scale bars, 100 µm (J).

Our previous studies have clarified the tumor-suppressive role of SIRT4 in HCC via mediating glutaminolysis. To examine whether SIRT4 exerts similar functions in breast cancer, we conducted LC-MS/MS analysis of metabolites in mammary epithelial cells from *SIRT4^WT^* and *SIRT4^-/-^* mice (n = 6). Consistently, glutamine was significantly up-regulated by SIRT4 deficiency (Figure 7A).

Moreover, SIRT4-depleted mouse mammary gland cells exhibited significantly increased glutamine uptake and NH4^+^ production (Figure 7B). Importantly, glucose uptake and lactate production were not affected, indicating that SIRT4 loss increased the ability of cells to utilize glutamine for mitochondrial energy production, as addressed by other studies 17. Similar observations were detected in SIRT4-knockdown human MDA-MB-468 and MCF-7 cells (Figure S14A-B). Conversely, forced expression of wild-type (but not catalytic H161Y mutant) SIRT4 in MDA-MB-231 and BT549 cells led to decreases in glutamine uptake and NH4 + production. However, it was not observed in those cells overexpressing catalytically inactive SIRT4 H161Y mutation (Figure S14C-D). Collectively, these data indicated that SIRT4 depletion affected glutaminolysis in mammary tumor cells.

In our previous study, we found that as a mitochondrial sirtuin, SIRT4 could exert its tumor-suppressive function in HCC by inhibiting glutamine metabolism and thereby regulating AMP-activated protein kinase (AMPK) /mTOR Axis 15. AMPK, a highly conserved sensor of cellular energy and nutrient status, has been reported to phosphorylate SIRT1 and inhibit its deacetylation activity in lung cancer and HCC 29, 30. Given the negative regulation of SIRT4 on SIRT1 and the intricate relationship between AMPK and SIRT1, we hypothesized that glutamine metabolism disorder might be involved in SIRT4-induced SIRT1 inhibition. To test this hypothesis, we treated the cells with bis-2-(5-phenylacetoamido-1,2,4-thiadiazol-2-yl)ethyl sulfide (BPTES) or 968, inhibitors of glutaminase 1 (GLS1), which is the first studied enzyme necessary for mitochondrial glutamine metabolism and its inhibition limits entry of glutamine flux into the TCA cycle 31-33. Treatment of BPTES or 968 successfully reversed the altered protein levels by SIRT4 deficiency, including SIRT1 and its downstream H4K16ac and BRCA1 as well as SOX2 (Figure 7C-D; Figure S15A). Likewise, SP cells, sphere formations, and CD24^-^CD44^+^ populations induced by SIRT4 depletion were also abrogated by GLS1 inhibition (Figure 7E-G; Figure S15B-D). Specifically, overexpression of SIRT4 in MDA-MB-231 and BT549 cells triggered phosphorylation of AMPKα, which in turn inhibited SIRT1. In contrast, AMPKα knockdown almost completely recovered the expression of SIRT1, suggesting the essential role of AMPKα in SIRT4-modulated expression of SIRT1 (Figure S15E-F). *In vivo*, evidence showed similar results that BPTES treatment successfully reversed the tumor-promoting effects by SIRT4 deficiency (Figure 7H-J). Together these results signified that SIRT4-deletion enhanced metabolic flux into glutaminolysis of mammary cells in conjunction with phosphorylation of AMPKα and the subsequent inhibition of SIRT1 and its downstream targets.

### EX-527 significantly eliminates SIRT4 depletion-induced BTICs and xenograft formation

EX-527 or Selisistat, a highly potent and selective inhibitor of SIRT1, used to being developed as a disease-modifying therapy for Huntington's Disease 34. With the accumulated evidence about SIRT1's function in cancer, EX-527 and other SIRT inhibitors have been studied as potential therapies in multiple cancer cells, including melanoma 35, hepatocellular carcinoma 36, chronic lymphocytic leukemia 37, and lung cancer 38. Despite an inhibitory or synergistic suppressive effect observed in the cells, *in vivo* confirmative observations are missing. Concerning breast cancer, EX-527 has been implicated in chemoresistance in several studies when being combined with different drugs 39-41.

**Figure 8:**
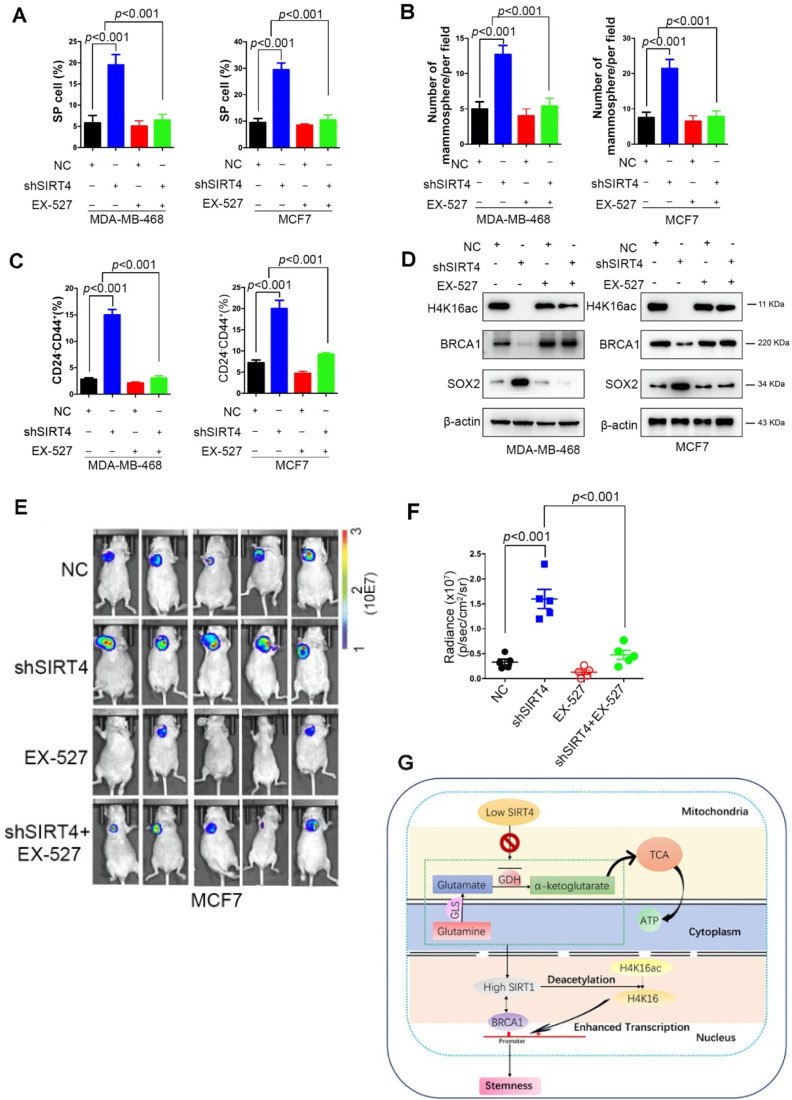
** EX-527 significantly eliminates SIRT4 depletion-induced BTICs and xenograft formation.** (**A, B, C**) Quantification of Hoechst SP assay (A), sphere formation efficiency (B), and CD44^+^/CD24^-^ subpopulations (C) in transformed MDA-MB-468 (left) and MCF-7 cells (right panel) described in Fig. 3G with or without treatment of EX-527, a highly potent and selective inhibitor of SIRT1. (**D**) Immunoblotting of indicated proteins isolated from transformed MDA-MB-468 (left) and MCF-7 cells (right panel) described above with or without EX-527 treatment. (**E, F**) Representative ventral view images of bioluminescence from mice with injections of cells described above (E) and its quantification (F). (**G**) A schematic model was illustrating the biological processes regulated by SIRT4 in breast cancer. Data are means ± SEM. *p* < 0.01; *t*-test.

To explore the potential clinical significance of EX-527, MDA-MB-468, and MCF-7, cells were treated with or without EX-527 at 38 nM dose. Not surprisingly, EX-527 treatment eliminated the increase of SP cells, mammosphere formation, and CD24^-^CD44^+^ populations induced by shSIRT4 in both cell lines (Figure 8A-C). Coincident observations were achieved by immunoblotting and immunofluorescence assay that SIRT1 inhibition completely abrogated the alterations of SIRT4 loss on the potential downstream targets, H4K16ac, BRCA1, and SOX2 (Figure 8D). *In vivo* evidence from mouse xenografts injected with transformed cells with or without EX-527 treatment revealed that SIRT1 inhibition counteracted the effects of SIRT4 decline on tumor growth (Figure 8E-F). Collectively, these data suggest that EX-527 might be an option for breast cancer patients with low SIRT4 expression.

## Discussion

The studies on SIRT4 have been increasingly reported during the past few years; however, SIRT4 remains to be the least well-characterized sirtuin. The mitochondrial SIRT4 is intimately involved in metabolic regulation with the inhibitory effect against insulin secretion in pancreatic β cells or fatty acid oxidation in liver and muscle cells 42, 43. Consistent with the importance of mitochondrial metabolism in tumorigenesis, SIRT4 was found to mainly exert tumor-suppressive function under its metabolic regulatory role 44. SIRT4-mediated inhibition of glutamine catabolism has been implicated in DNA damage response, cell proliferation, migration, and invasion in multiple cancers 15, 17, 45. In terms of breast cancer, low SIRT4 expression was linked with poor overall survival in breast cancer patients, especially in Luminal A patients 21. Consistent with this finding, we also revealed that the correlation between decreased *SIRT4* expression and increased risk of disease progression. To the best of our knowledge, no direct role of SIRT4 in regulating reprogramming or stemness has been reported. Correctly, SIRT4 has been shown to prevent the senescence of somatic cells, which might facilitate the reprogramming 46, 47. Here, with *SIRT4*^-/-^ mice and crossed *MMTV-neu*;* SIRT4*^-/-^ mice, we identified an unrecognized role of SIRT4 in regulating mammary gland development and stemness, contributing to mammary tumorigenesis. This result was further validated by the RNA-seq and GO analysis of genes significantly differentially expressed in response to SIRT4 knockout. Breast tumor initiating cells can be enriched by sorting for CD24^-^CD44^+^ cells, by selecting for side-population cells that efflux Hoechst dyes, or by isolating mammospheres from suspension cultures 48-50. Consistently, ablation of SIRT4 caused an evident expansion while SIRT4 overexpression inhibited self-renewal ability of these BTICs.

The complex system of interactions mediated by mammalian sirtuins is mostly involved in many metabolic processes as well as in different diseases. The cellular distribution and biological functions of these proteins are different though; different sirtuins also share a conserved catalytic domain and control similar cellular processes, suggesting a co-ordinated mode of action 47, 51. While there is a substantial knowledge of the biological functions of sirtuins, studies about the interplay and potential cross-regulation within this family are still limited. Recently, the SIRT1-SIRT3 axis has been identified to regulate the cellular response to oxidative stress and etoposide. SIRT1-silencing increases SIRT3 promoter activity depending on the presence of SP1 and ZF5 recognition sequences on SIRT3 promoter 52. Besides, SIRT1 was shown to regulate SIRT3 expression after oxygen and glucose deprivation through inhibiting the AMPK-PGC1 pathway, which might be an essential modulator in blood-brain barrier physiology 53. To efficiently deacetylate targets such as p53, H3K9, and H4K16, SIRT1 requires autodeacetylation to be activated, which could be restricted by SIRT7 during adipogenesis in mice 54. The protein-protein interaction network of the human sirtuin family showed that SIRT4 was interacting with all the sirtuin family, including SIRT1 55, which is also confirmed by our proteome-wide analysis of SIRT4 deficiency-induced expression in mouse mammary gland cells. Our data illustrate that SIRT4 negatively regulates SIRT1 expression in breast tumors.

Mechanistically, SIRT4 was known to attenuate the activation effect of SIRT1 on PPARα transcription by interfering with the binding of SIRT1 with PPARα response elements and thus represses hepatic fatty acid oxidation 56. In this study, we demonstrate that SIRT4 activity dampens breast cancer stemness by modulating SIRT1 expression, as supported by the evidence that genetic and pharmacological inhibition of SIRT1 expression or activity could successfully eradicate the expansion of BTICs or xenograft formation induced by SIRT4 loss. More importantly, the metabolomic analysis identified glutamine metabolism disorder in mammary gland cells from *SIRT4*^-/-^ mice, which is consistent with the metabolic regulatory role of SIRT4 in glutamine uptake and utilization. GLS1 inhibitors, BPTES, or 968 blunts the xenograft formation and increase in SIRT1 protein levels as well as the BTIC phenotype observed in SIRT4 null cells. Our findings thus highlight the unique effect of SIRT4 on suppressing the expression and function of SIRT1 through modulating glutamine metabolism, providing a novel cross-talk between mitochondrial and nuclear sirtuins.

As the most commonly diagnosed cancer in women worldwide, breast cancer is a multifactorial genetic disease. 85 to 90% of breast tumors are sporadic, which are uniquely characterized by an altered epigenome. Deregulated histone epigenome, along with other epigenetic alterations play a vital role in the initiation and progression of breast cancer. Global loss of H4K16ac and under-expressed H3K9ac is a hallmark of breast cancer, the expression pattern of which could be modulated by SIRT1 through direct deacetylation 27, 57. Moreover, the colocalization of SIRT1 and its H3 acetylated targets was identified on the *BRCA1* promoter in a subtype-specific manner 27. So far, no direct evidence has linked SIRT4 to modulation of H4K16ac and BRCA1 expression. Until very recently, the first SANT domain (SANT1) of BRCA1 was reported to be a histone binding domain with specificity for the histone H4 N-terminal tail. Acetylation of H4K16 consequently abolishes binding of SANT1 to H4 58, suggesting potential regulation of BRCA1 expression by H4K16ac. Consistently, our ChIP-seq results uncovered that SIRT4 deficiency down-regulates BRCA1 expression through modulation of H4K16ac rather than the well-defined H3K9ac, which is further confirmed by following ChIP-qPCR assay. Furthermore, we also validated the two putative H4K16ac binding sites on Exon 1 of BRCA1, which has not been reported before.

## Conclusions

In conclusion, we found that SIRT4 regulation of BITCs negatively modulated mammary tumorigenesis through suppression of SIRT1 via glutamine metabolism and that SIRT4 deficiency decreased H4K16ac and BRCA1 expression in breast cancer (Figure 8G). These findings shed light on novel molecular mechanisms of glutamine metabolism modulation by SIRT4 and cross-talk between mitochondrial and nuclear sirtuins, suggesting that SIRT4 has tumor-suppressive activity and may serve as a novel therapeutic target in breast cancer.

## Supplementary Material

Supplementary figures.

## Abbreviations

AMPK: AMP-activated protein kinase
bFGF: Basic fibroblast growth factor
BPTES: Bis-2-(5-phenylacetoamido-1,2,4-thiadiazol-2-yl)ethyl sulfide
BTICs: Breast tumor-initiating cells
ChIP-seq: Chromatin immunoprecipitation/high-throughput sequencing
EGF: Epidermal growth factor
GEO: Gene expression omnibus
GLS1: Glutaminase 1
GSEA: Gene set enrichment analysis
HDACs: Histone deacetylases
MC: methylcellulose
MEGM: Mammary epithelial growth medium
MG: Matrigel
MSC: Mammary stem cells
NAD+: Nicotinamide adenine dinucleotide
SIR2: Silent information regulator 2
SP: Side population
SRM: Selected reaction monitoring
TCGA: The cancer genome atlas
TEB: Terminal end bud
TICs: Tumor-initiating cells
