# Supplementary material for: Loss of SIRT4 promotes the self-renewal of Breast Cancer Stem Cells
Source: Theranostics. 2020 Jul 25;10(21):9458–76. doi: 10.7150/thno.44688 (PMC7449925; doi:10.7150/thno.44688)

Supplementary Figures and Figure legends

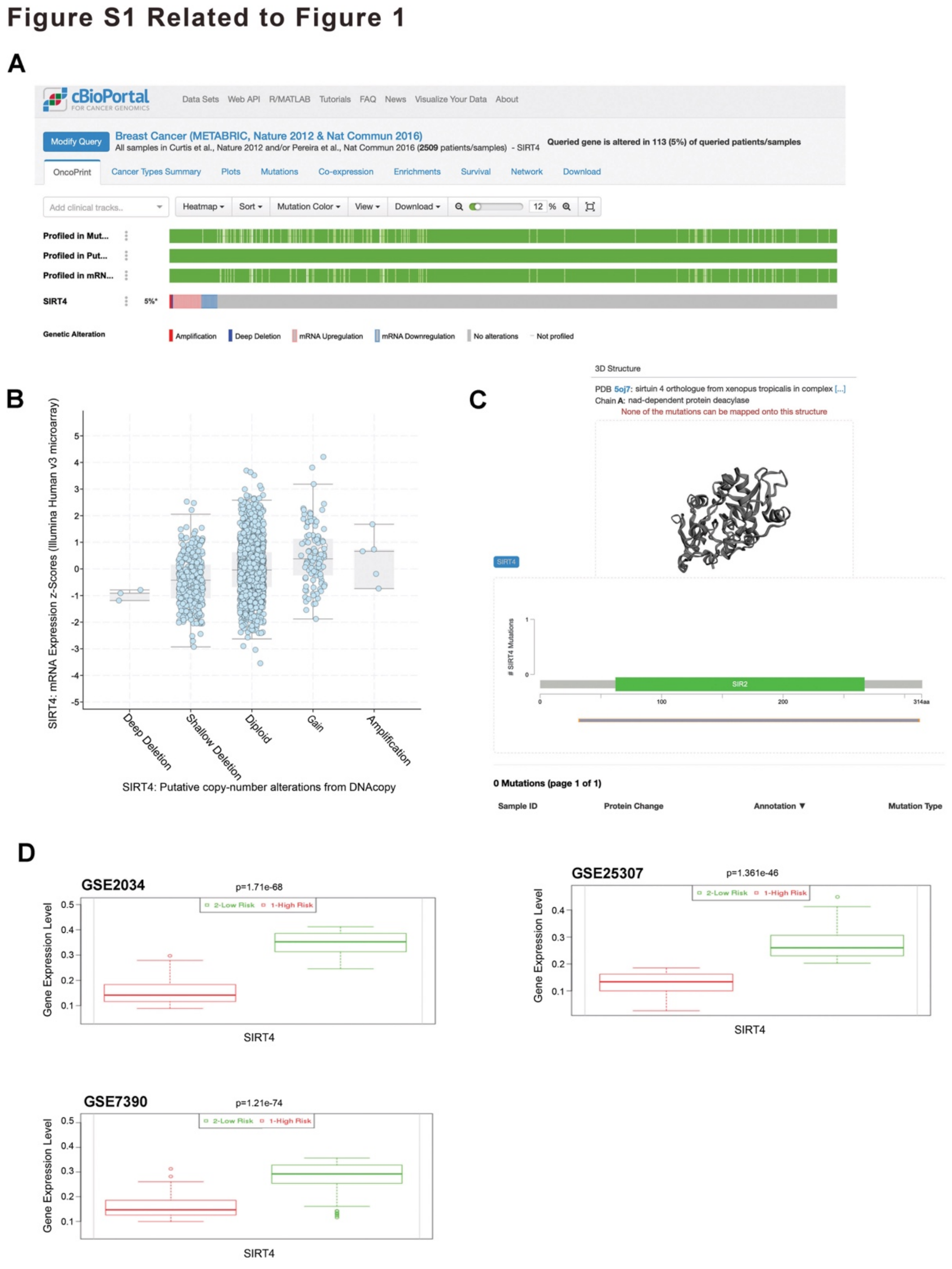

Figure S1. (A) TCGA database available from cBioportal for SIRT4 expression in human breast cancers. **(B)** Copy number compared to gene expression for SIRT4 mRNA versus copy number in human breast cancers using the cBioportal database. **(C)** Putative 3D structures of SIRT4 protein generated from the cBioportal database. **(D)**

Gene expression of SIRT4 in human breast cancer compared to normal tissues from 3 Gene Expression Omnibus (GEO) data sets (GSE2034, 25307, 7390).

Figure S2 Related to Figure 1

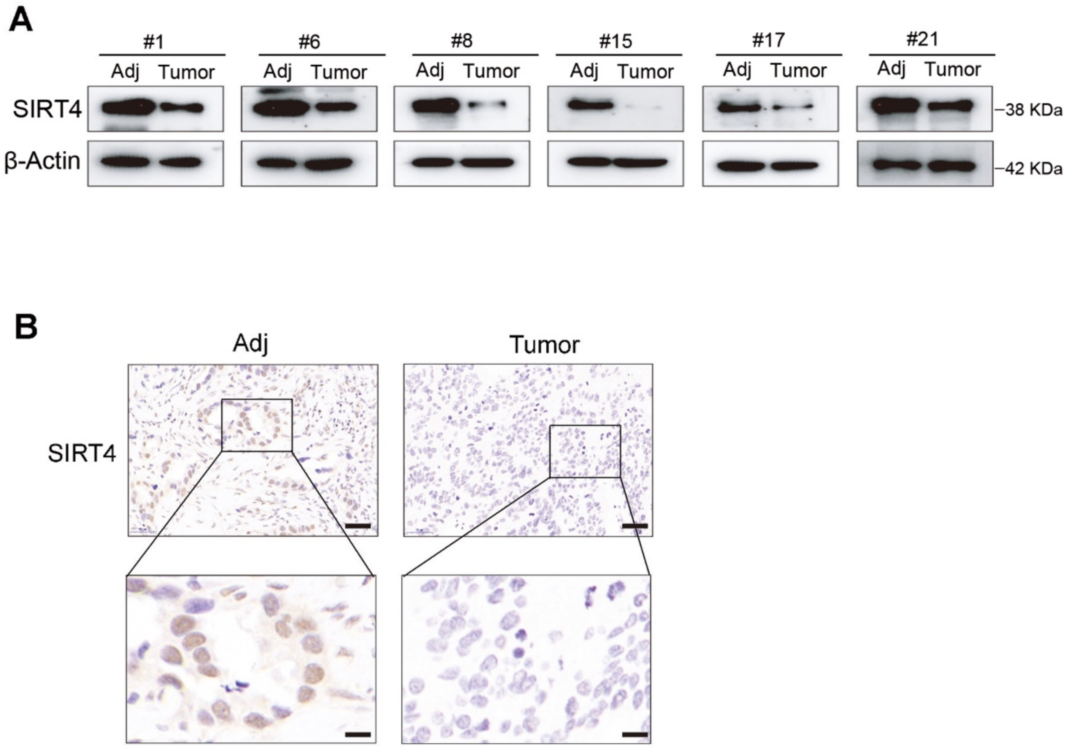

Figure S2. **(A)** Immunoblotting of SIRT4 in breast tumors compared to normal tissues (n=6). **(B)** Representative IHC staining images of SIRT4 in breast tumors compared to normal tissues; Scale bars, 100 μm (B) and 20 μm in the insets (B).

Figure S3 Related to Figure 1

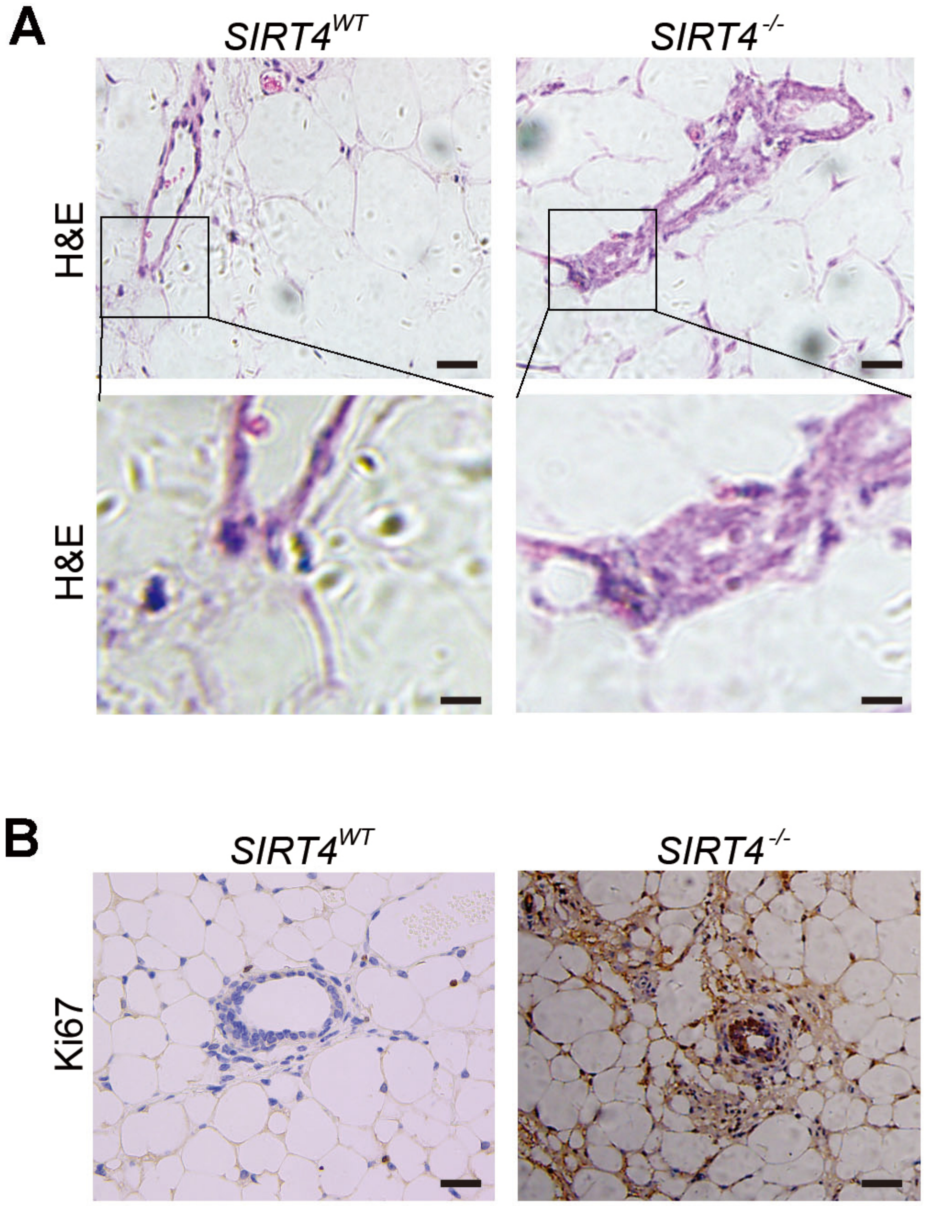

Figure S3. Representative H&E staining (A) and IHC staining images of Ki67 (B) for sections of mammary glands isolated from SIRT4^{WT} and SIRT4$^{-/-}$ mice; Scale bars, 100 μm (A) and 20 μm in the insets (A), 100 μm (B).

Figure S4 Related to Figure 2

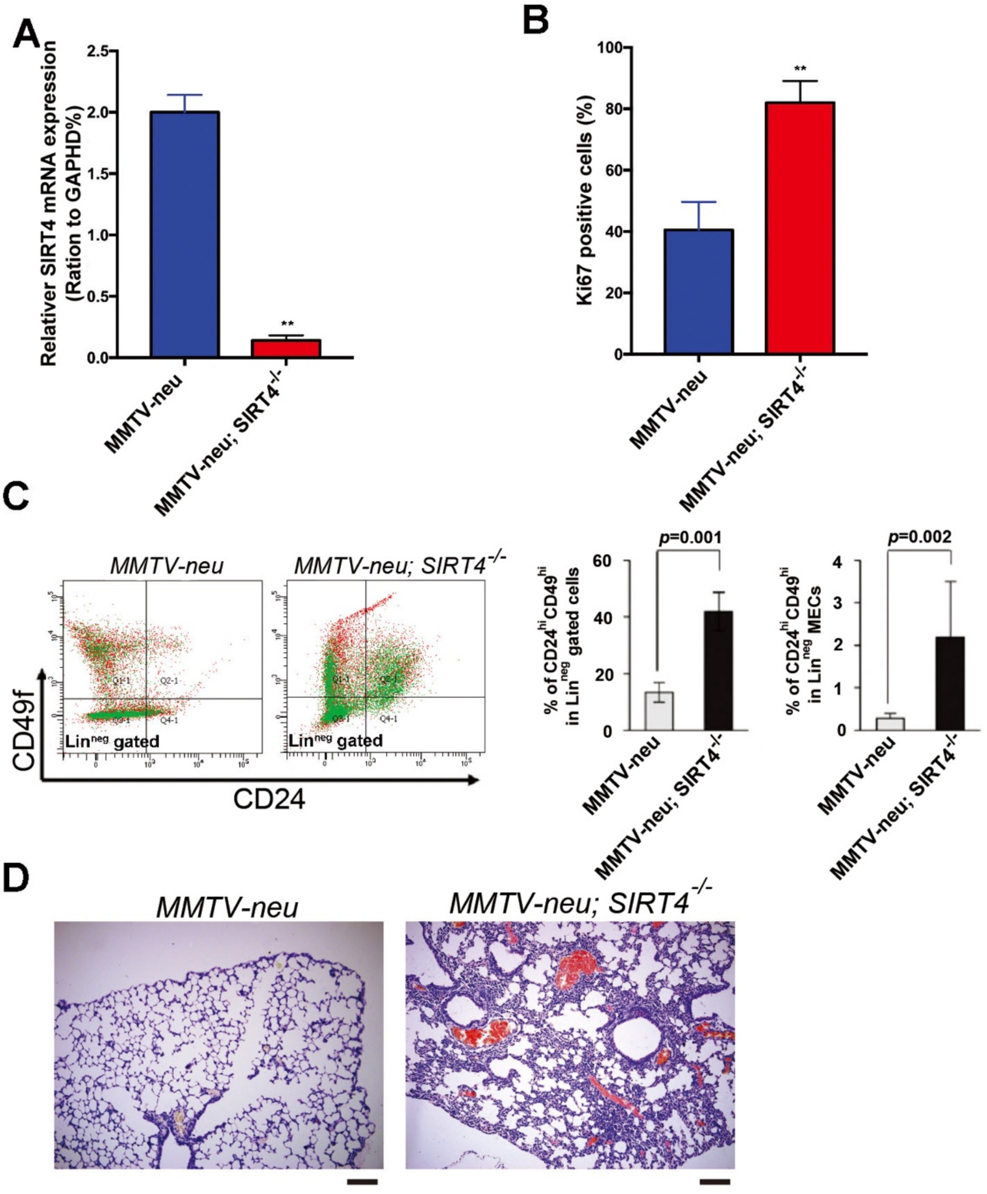

Figure S4. **(A)** mRNA expression of SIRT4 in tumors isolated from MMTV-neu and MMTV-neu; SIRT4$^{-/-}$ mice. **(B)** Quantification of Fig.2E. **(C)** Distribution of Linneg mouse mammary cells according to their expression of CD24 and CD49f were analyzed on MMTV-neu and MMTV-neu; SIRT4$^{-/-}$ mice (left) and its quantification (right). **(D)** Representative H&E staining images for sections of lung metastasis isolated from mice mentioned above; Scale bars, 200 µm (D).

Figure S5 Related to Figure 3

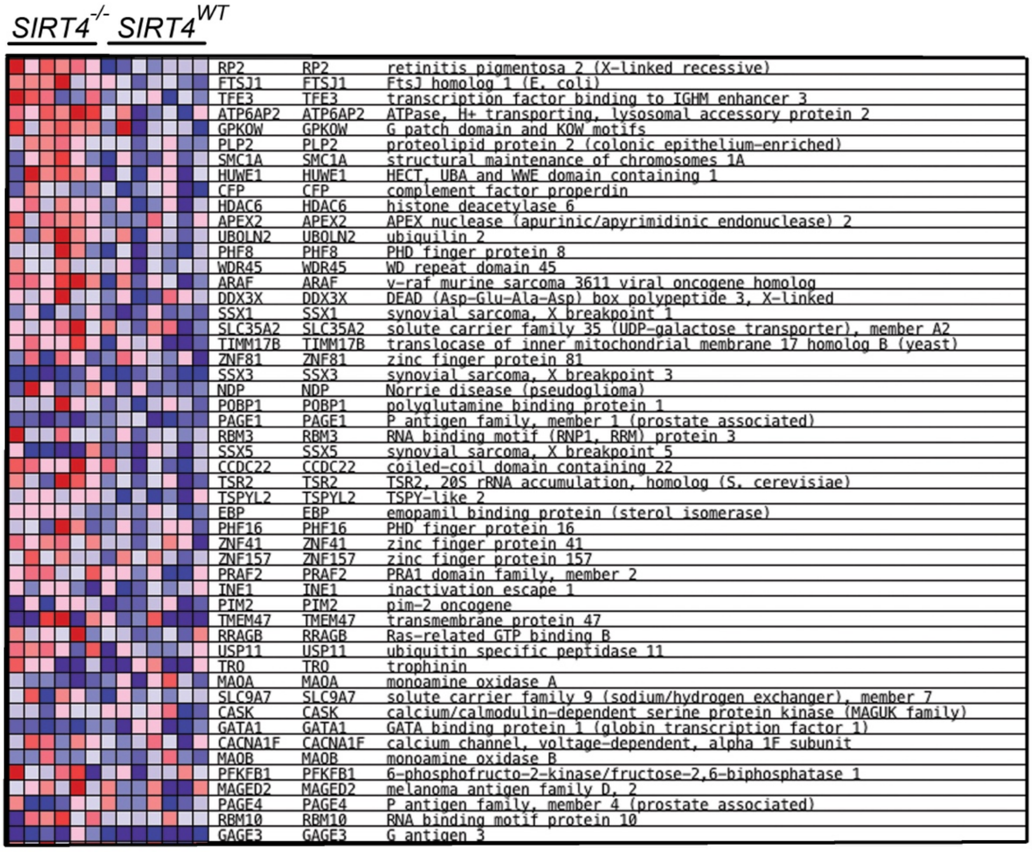

Figure S5. Top 50 of the genes displayed in Fig.3A.

Figure S6 Related to Figure 3

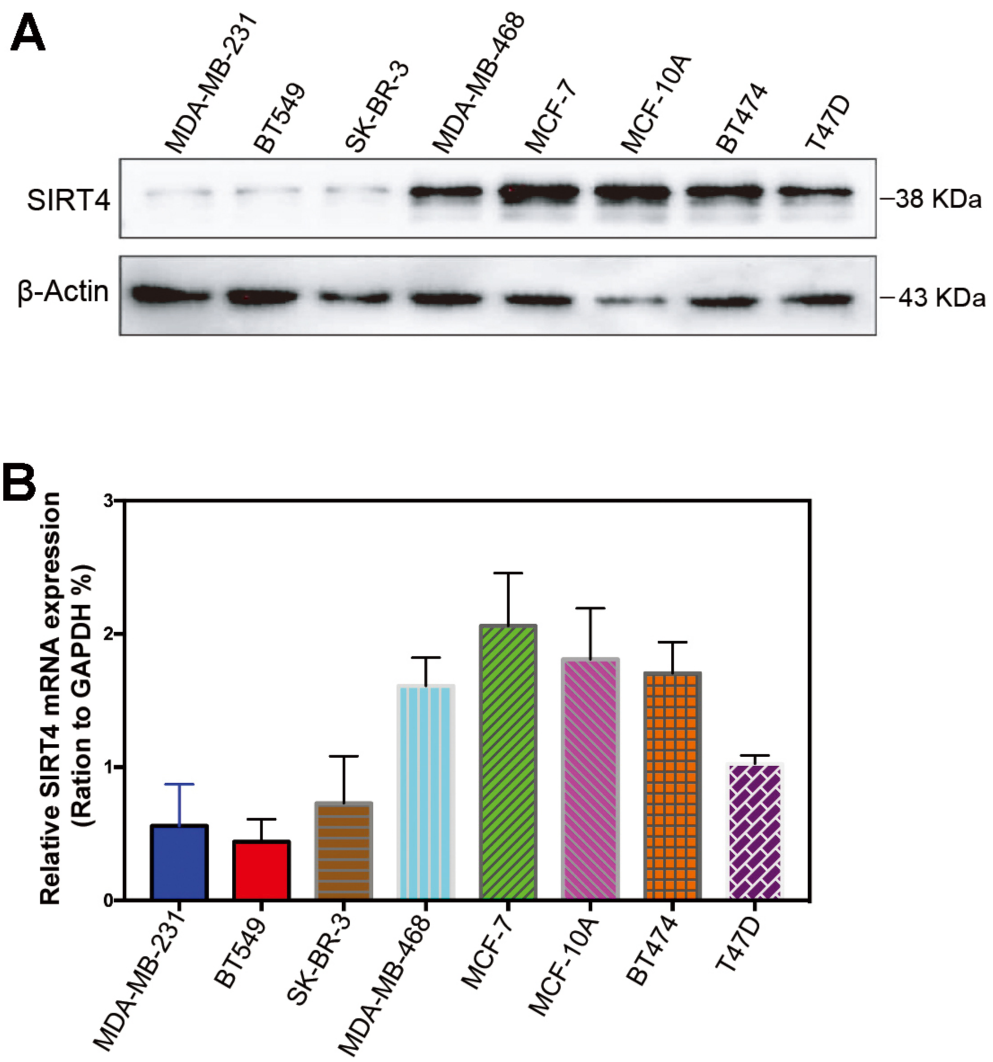

Figure S6. Immunoblotting (A) and mRNA expression (B) of SIRT4 among breast cancer cell lines.

Figure S7 Related to Figure 3

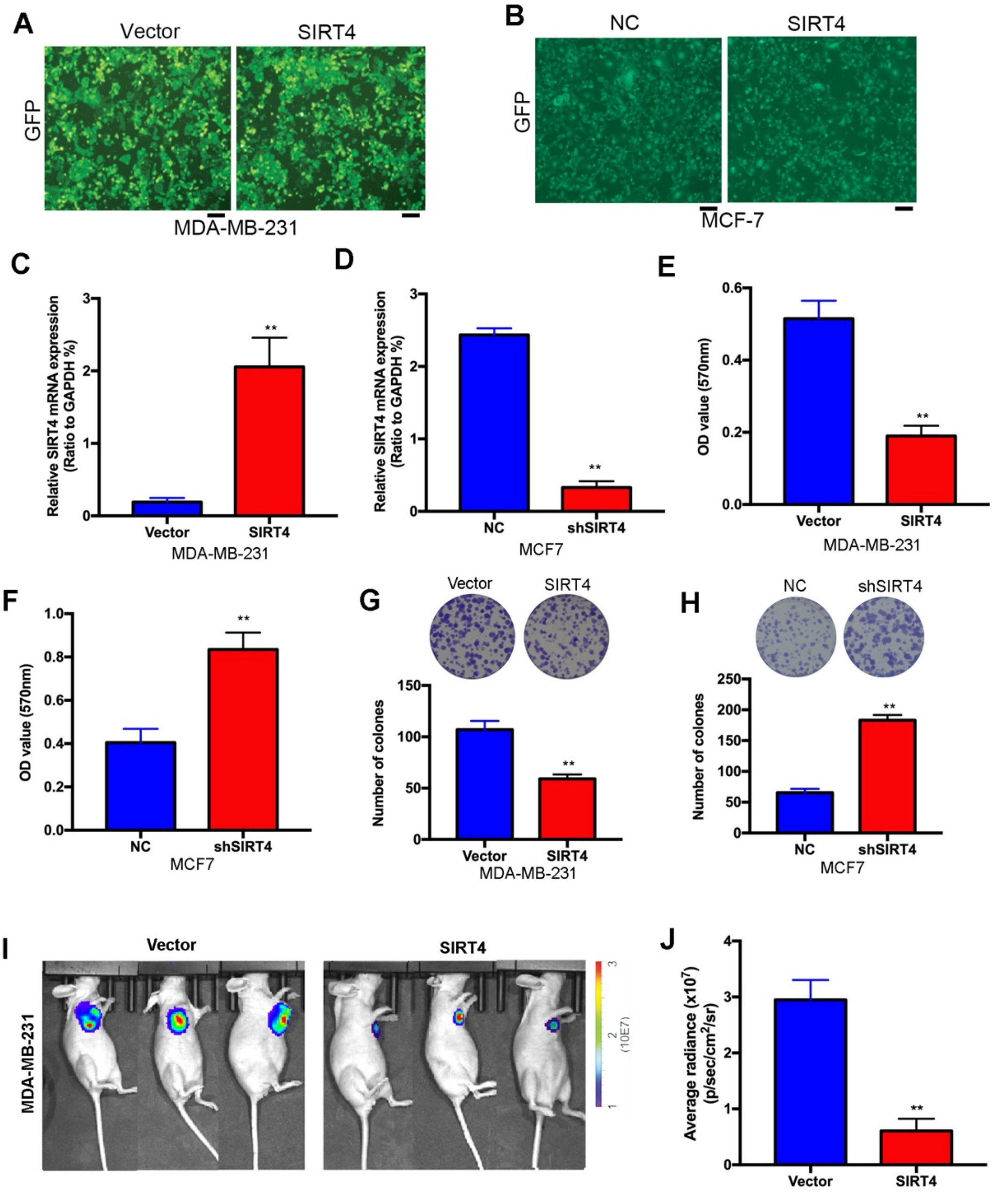

Figure S7. **(A-D)** Transfection efficiency of MDA-MB-231 (A, C) and MCF-7 cells (B, D) transfected with control vector, SIRT4, and/or sh-SIRT4 measured by GFP (A, B) or mRNA expression (C, D). **(E-H)** In vitro MTT (E, F) and sphere formation assay (G, H) with transformed MDA-MB-231 (E, G) and MCF-7 cells (F, H) described above. **(I, J)** Representative ventral view images of bioluminescence from mice (n=3) with injections of MDA-MB-231 cells described above (I) and its quantification (J).

Figure S8 Related to Figure 3

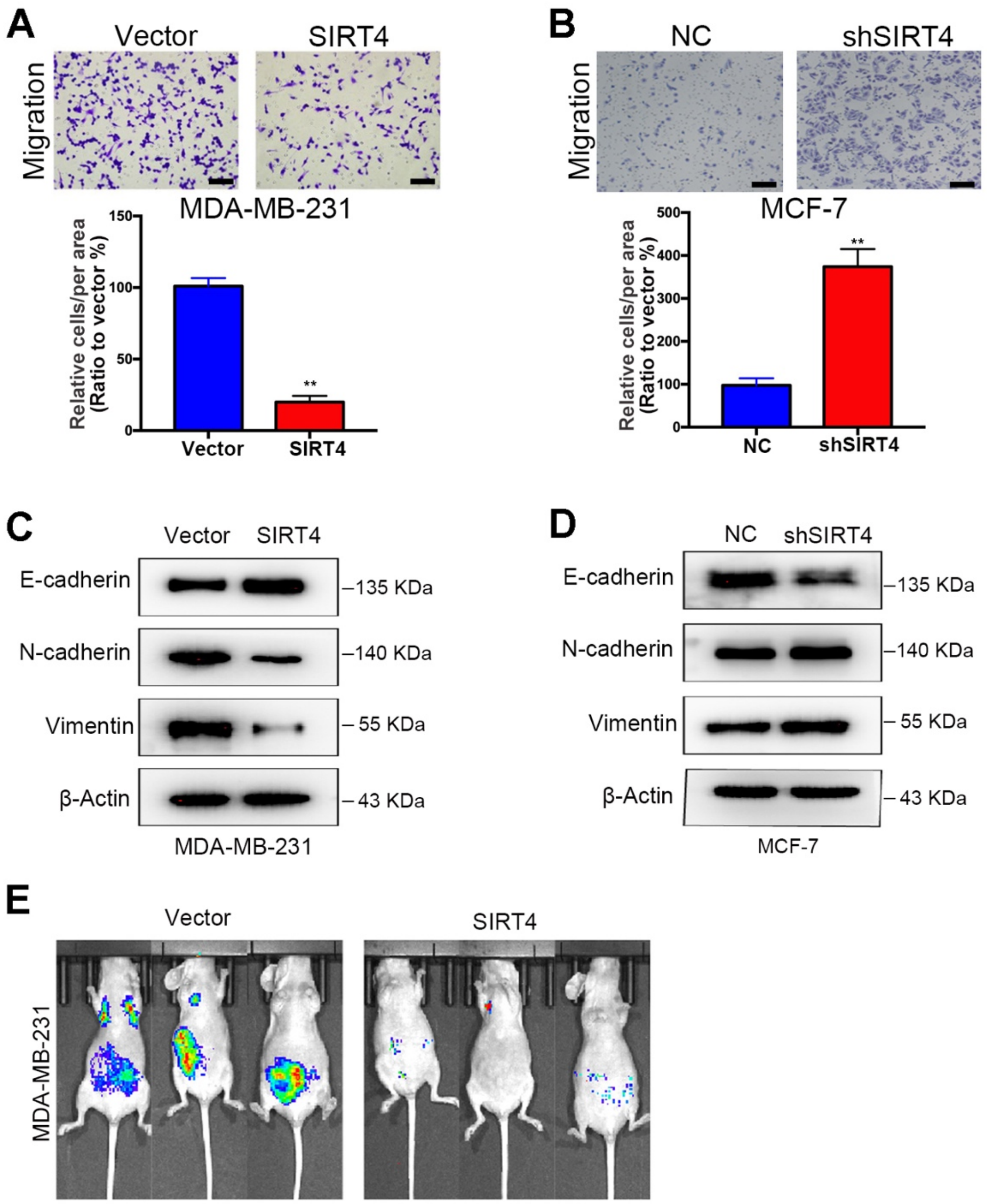

Figure S8. (A, B) Migration assay (upper) and its quantification (bottom) of transformed MDA-MB-231 (A) and MCF-7 cells (B) described in Supplementary Figure 8. (C, D) Immunoblotting of transformed MDA-MB-231 (C) and MCF-7 cells (D) described above. (E) Representative ventral view images of bioluminescence from mice (n=3) with injections of MDA-MB-231 cells described above; Scale bars, 100 μm (A) and 100 μm (B).

Figure S9 Related to Figure 4

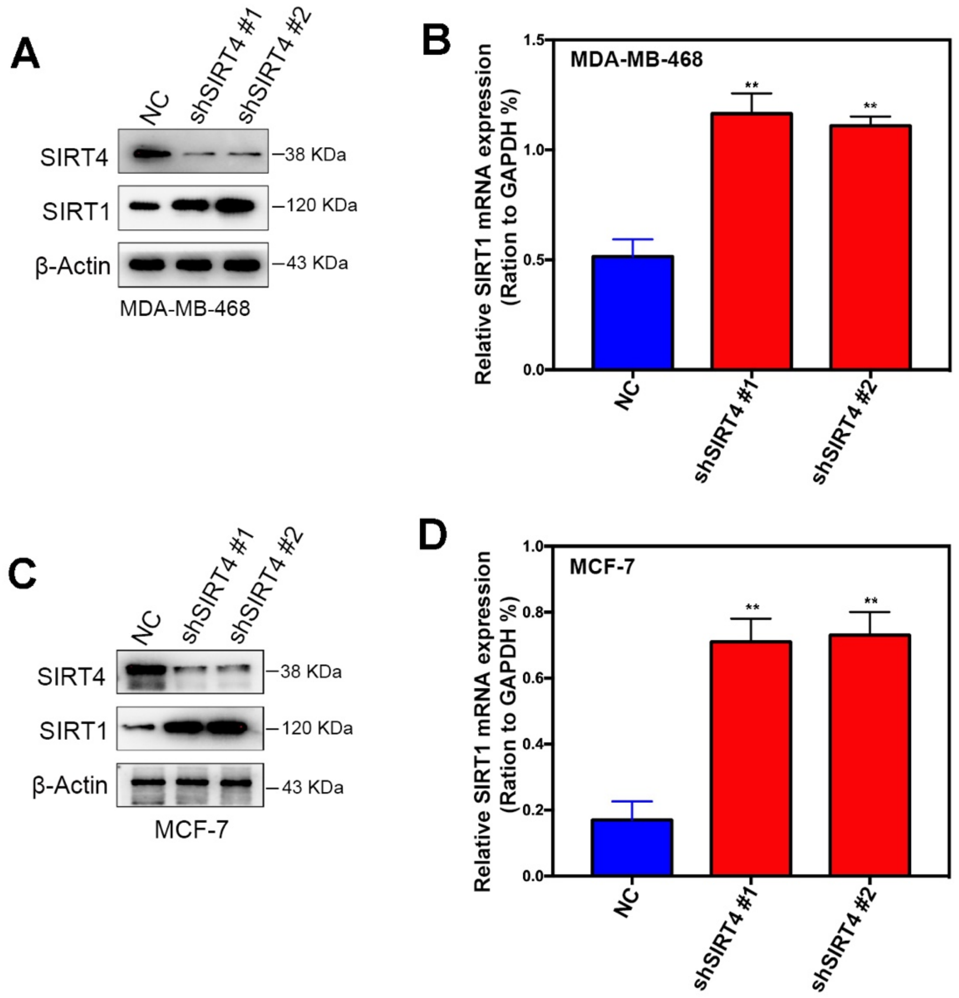

Figure S9. Immunoblotting (A, C) and mRNA (B, D) expression of SIRT4 and SIRT1 in transformed MDA-MB-468 (A, B) and MCF-7 cells (C, D) described in Fig.3G.

Figure S10 Related to Figure 4

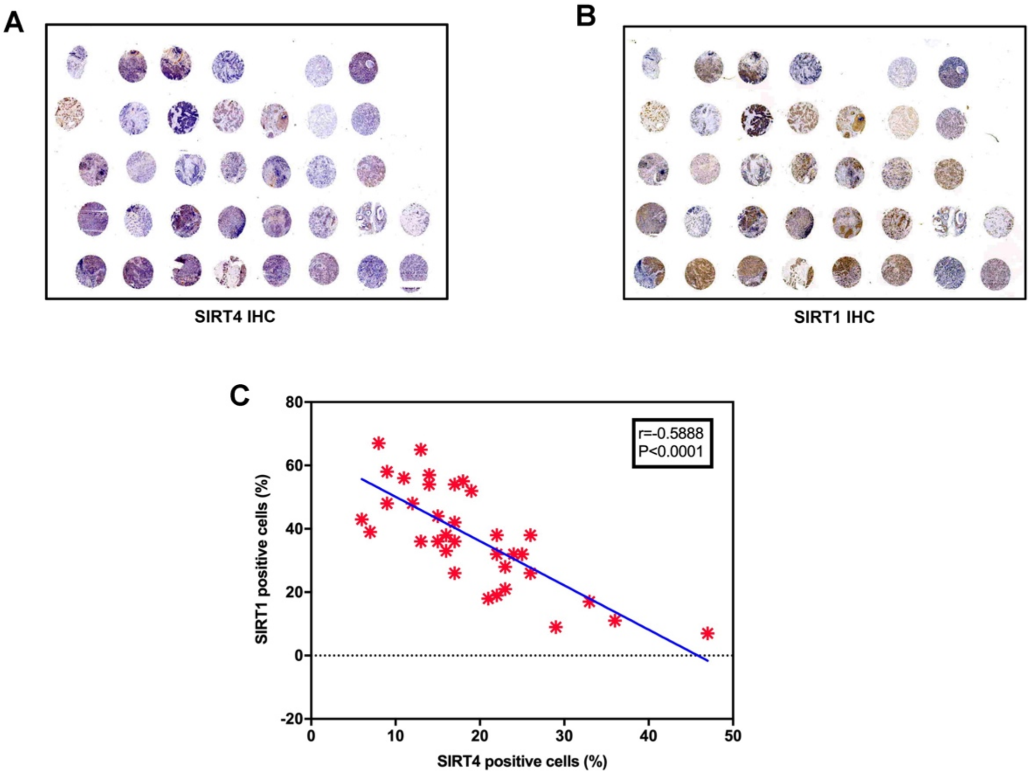

Figure S10. **(A, B)** Representative IHC staining images of SIRT4 (A) and SIRT1 (B) in mammary tissues from 36 breast cancer patients. (B) Quantification of (A, B). **(C)** Negative association between SIRT4 and SIRT1 protein expression in breast tumor tissues.

Figure S11 Related to Figure 5

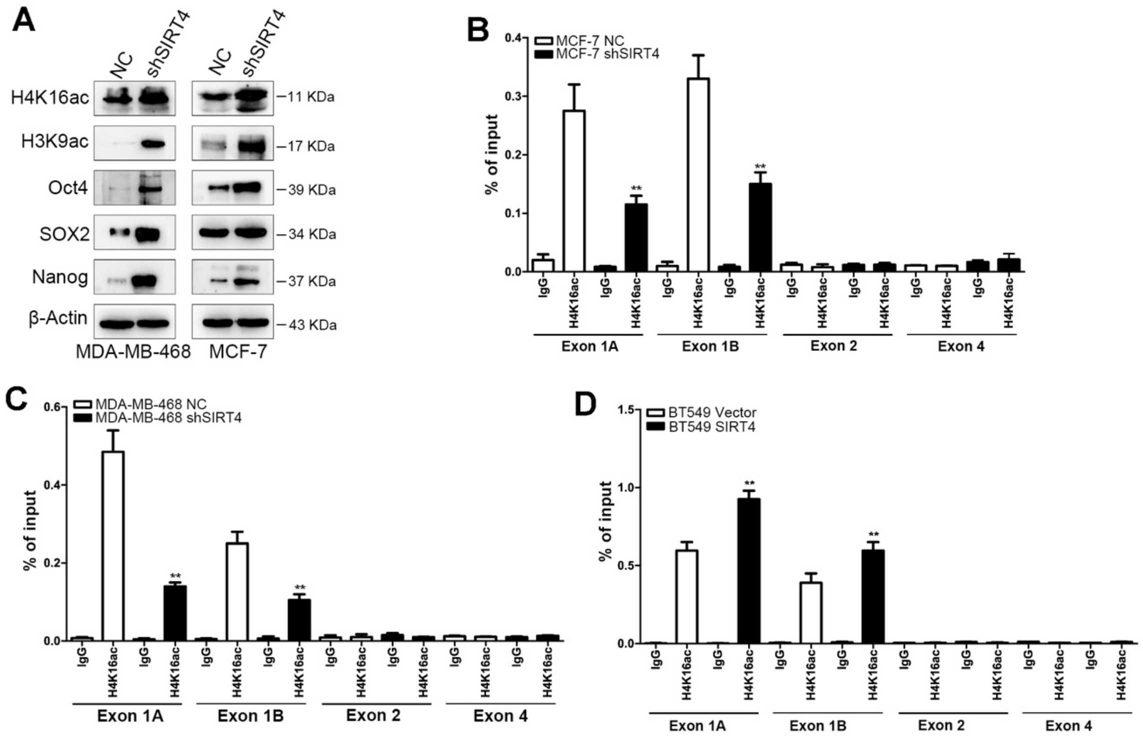

Figure S11. **(A)** Immunoblotting of acetyl-histone H4 at lys16 (H4K16ac), acetyl-histone H3 at lys9 (H3K9ac), Oct4, SOX2, and Nanog in MDA-MB-468 (left panel) as well as MCF-7 cells (right panel) transfected with control (NC) or shSIRT4 vector. **(B-D)** transformed MCF-7 (B), MDA-MB-231 (C), and BT579 cells (D) as described in Fig.3 were chromatin immunoprecipitated for IgG and H4K16ac. Pull-down at the putative H4K16ac binding sites was assessed by qRT- PCR and calculated as percent of IgG input. Error bars are SEM for 3 technical replicates.

Figure S12 Related to Figure 5

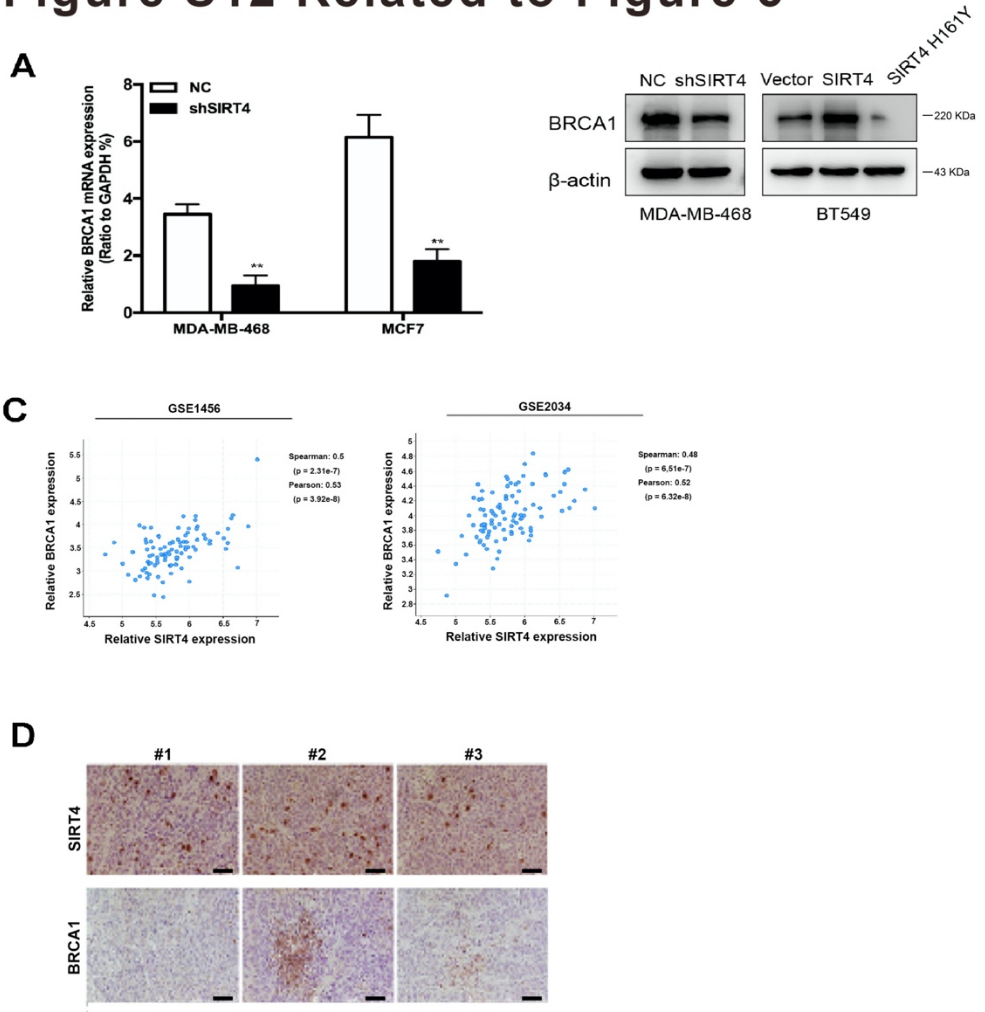

Figure S12. (A, B, C) Immunoblotting (B) and mRNA expression (C) of BRCA1 in MDA-MB-468, MCF7 and BT579 cells describe in Fig5. **(D)** Reverse relationship between SIRT4 and BRCA1 generated from 3 databases (GSE1456, 2034, and 4922). **(E)** Representative IHC staining images of SIRT4 and BRCA1 in tumor sections isolated from human mammary tissues; Scale bars, 100 μm (D).

Figure S13 Related to Figure 6

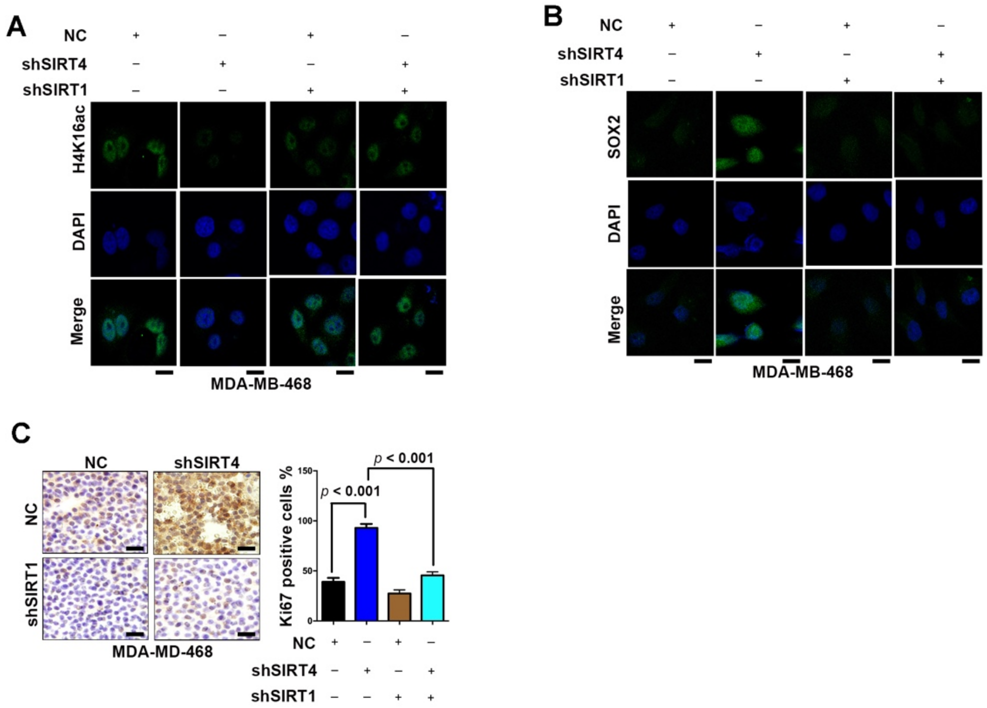

Figure S13. (A, B) Immunofluorescence images of MDA-MB-468 cells described in Fig.6A stained with antibodies against H4K16ac/DAPI (A) and SOX2/DAPI (B). **(C)** Representative IHC staining images (left) and its quantification (right) of Ki67 in MDA-MB-468 cells described above; Scale bars, 20 μm (A) and 20 μm (B).

Figure S14 Related to Figure 7

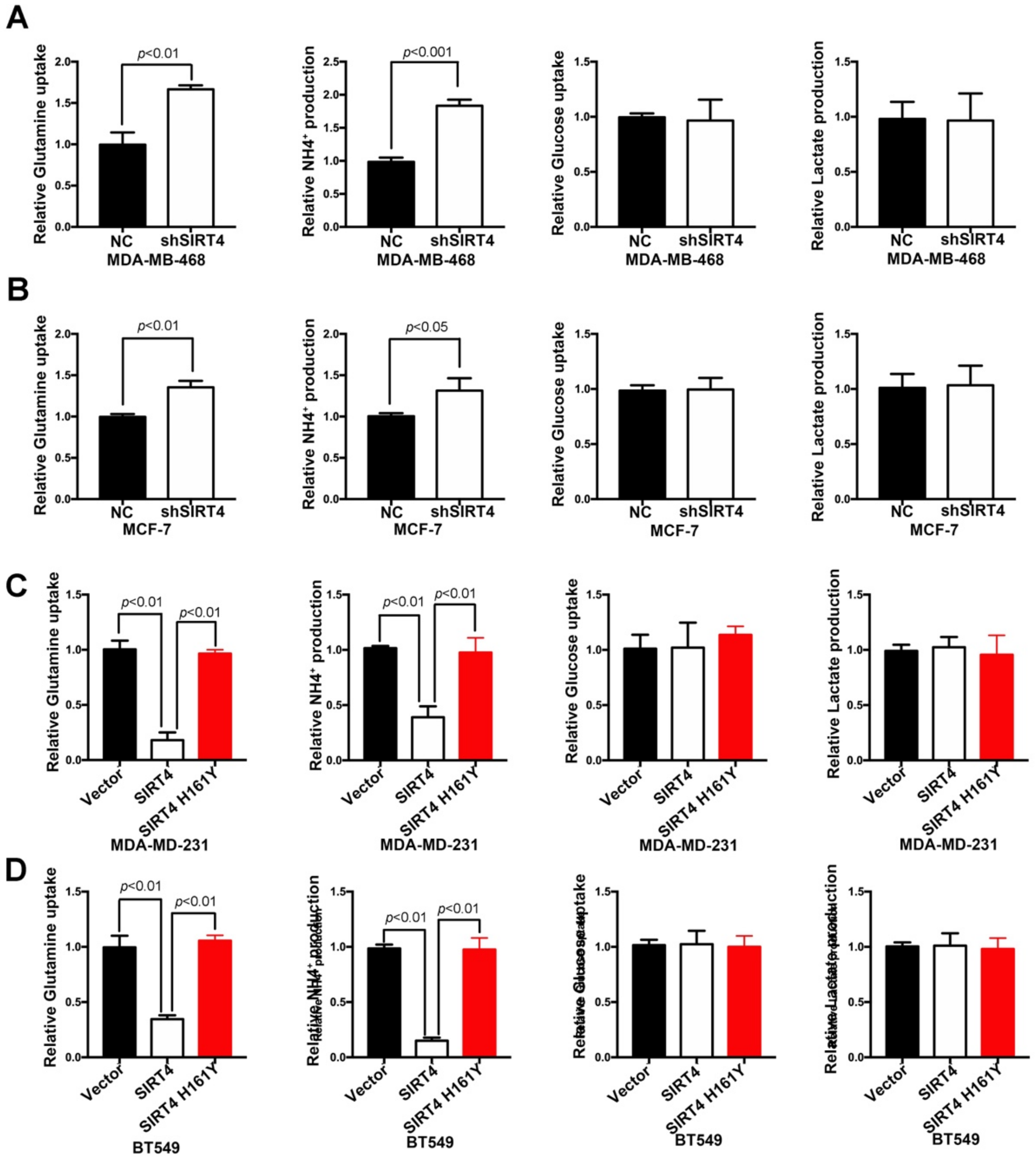

Figure S14. (A-D) Measurement of Glutamine uptake, NH4+ production, Glucose uptake, and lactate production (from left to right panel) in MDA-MB-468 (A), MCF-7 (B), MDA-MB-231 (C), and BT579 (D) cells.

Figure S15 Related to Figure 7

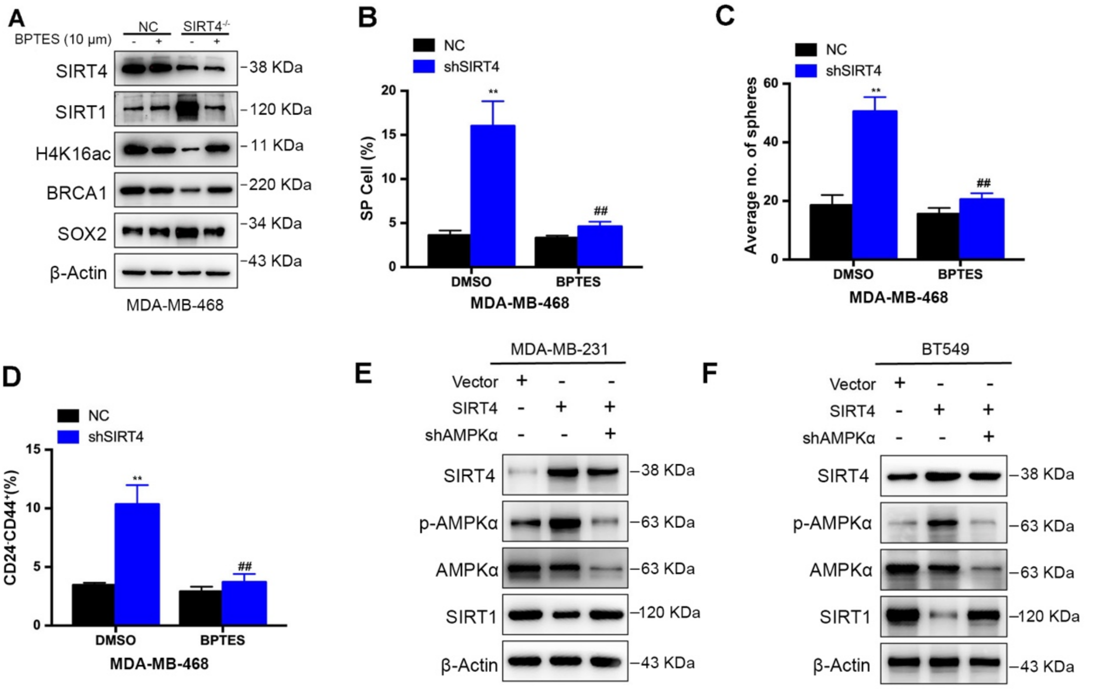

Figure S15. **(A)** Immunoblotting of indicated proteins isolated from MDA-MB-468 cells with or without BPTES (10 μM) treatment. **(B, C, D)** Quantification of Hoechst SP assay (B), sphere formation efficiency (C), and CD44+/CD24- subpopulations (D) in MDA-MB-468 cells with or without BPTES treatment.

Full unedited gel for western blots figures

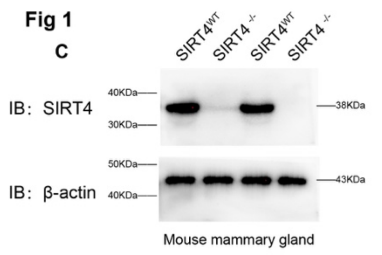

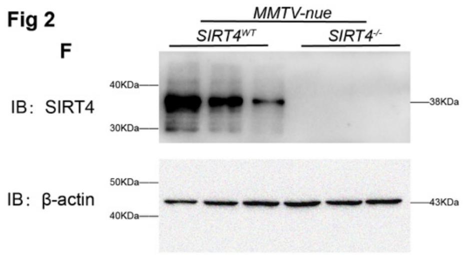

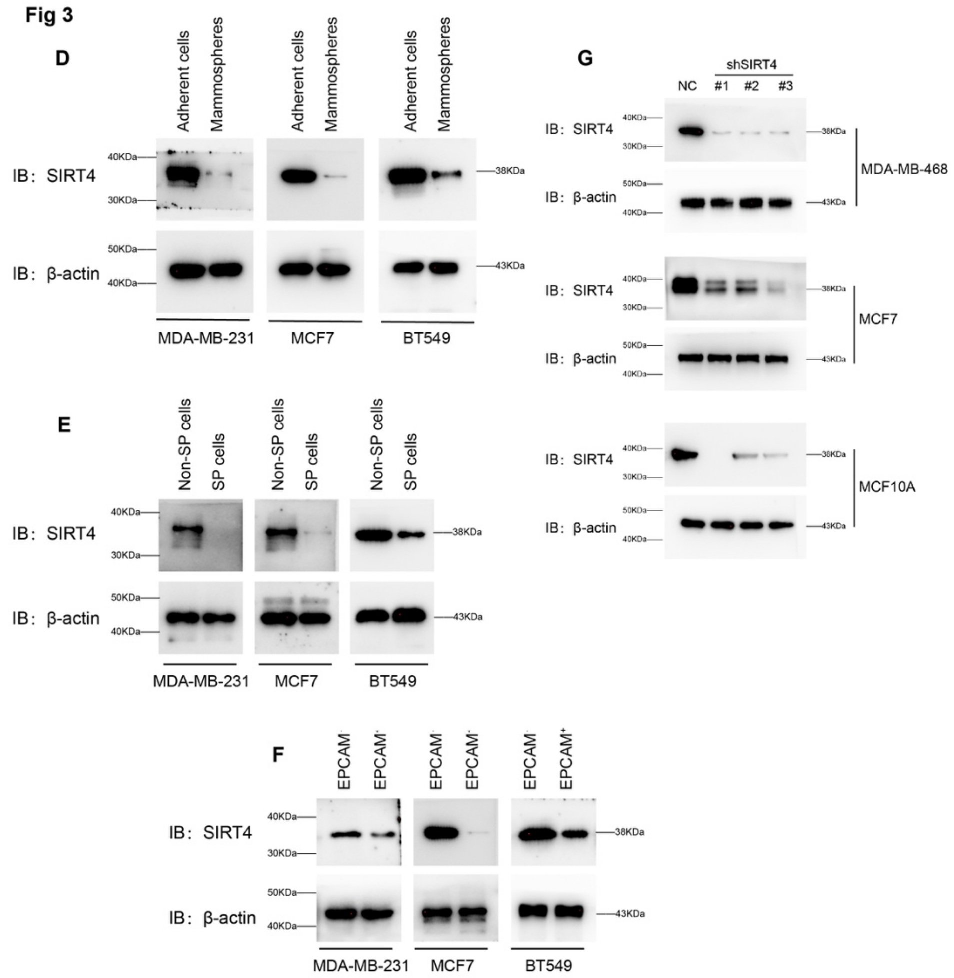

Fig 3

H

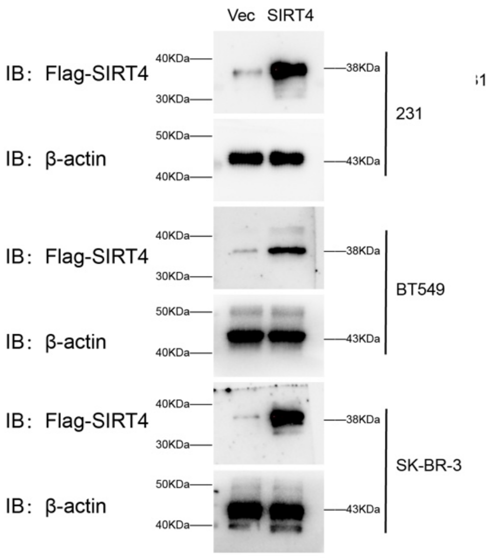

Fig 4

E

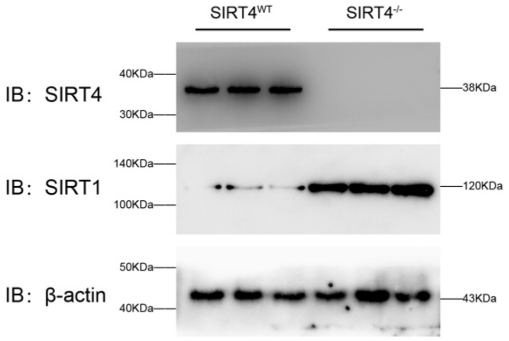

F

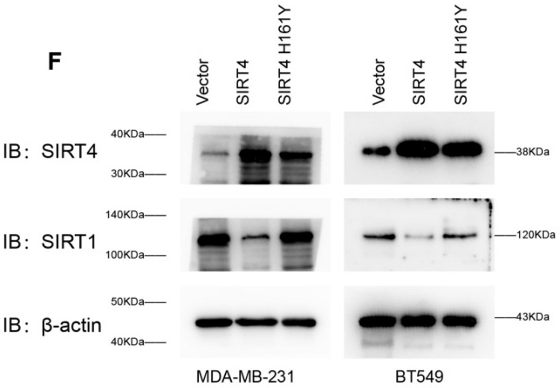

Fig 5

A

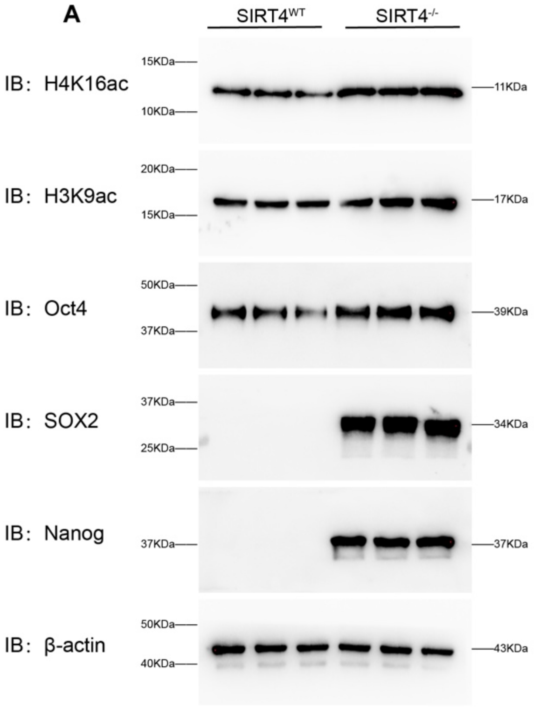

Fig 5

B

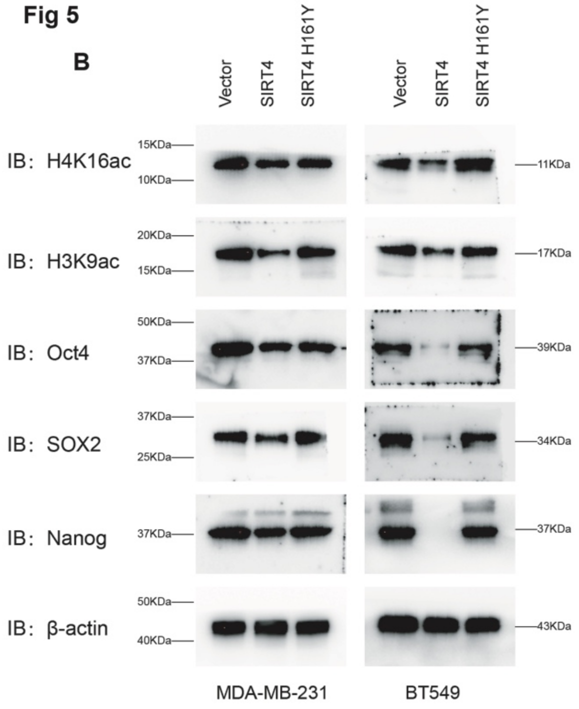

Fig 7

C

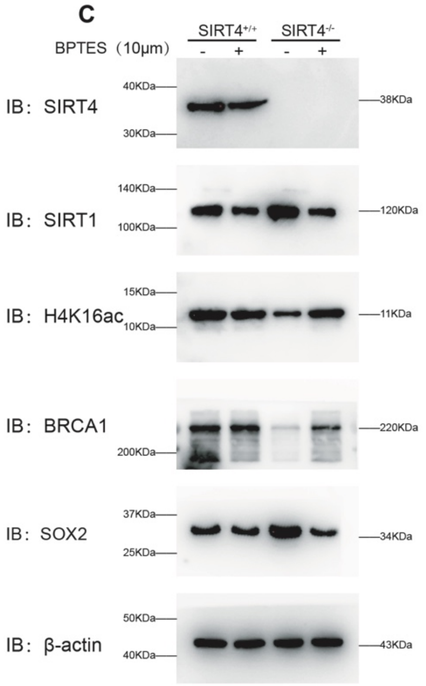

H

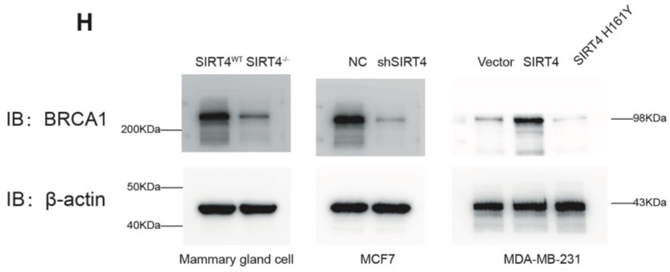

Fig 6

C

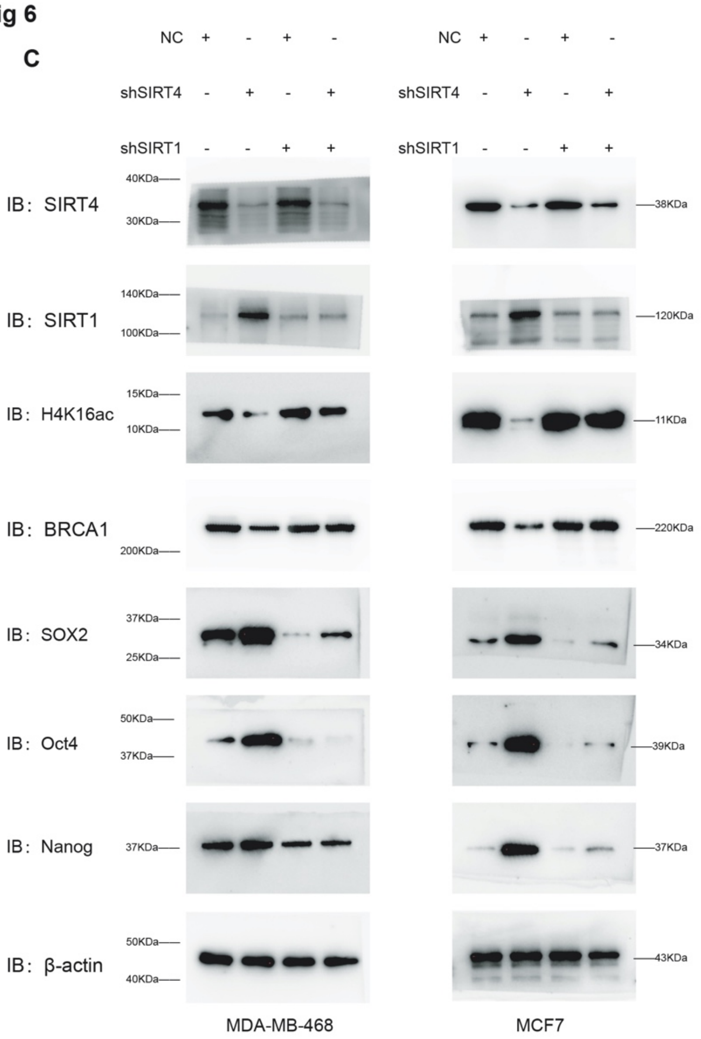

sFig 2

A

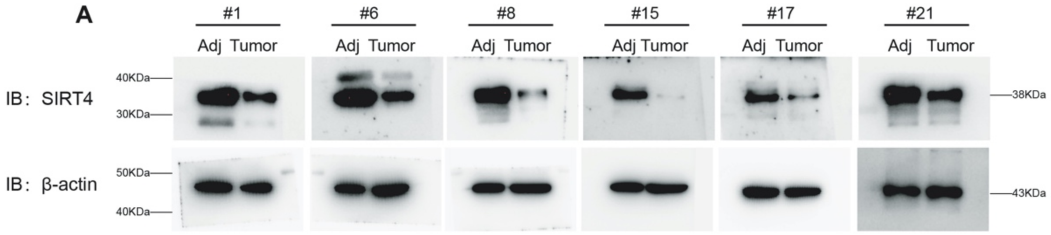

Fig 7

D

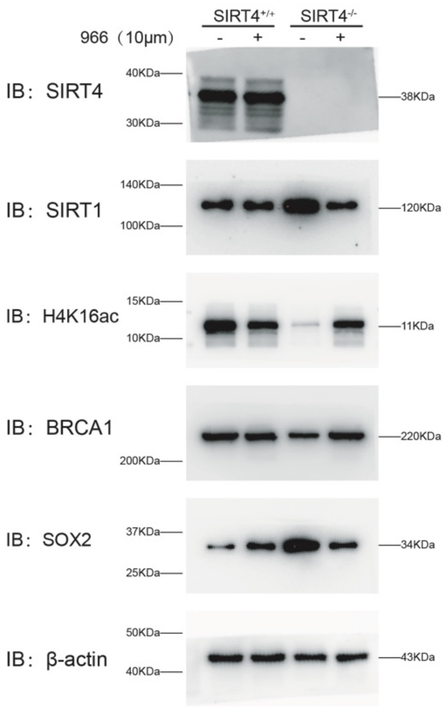

Fig 8

D

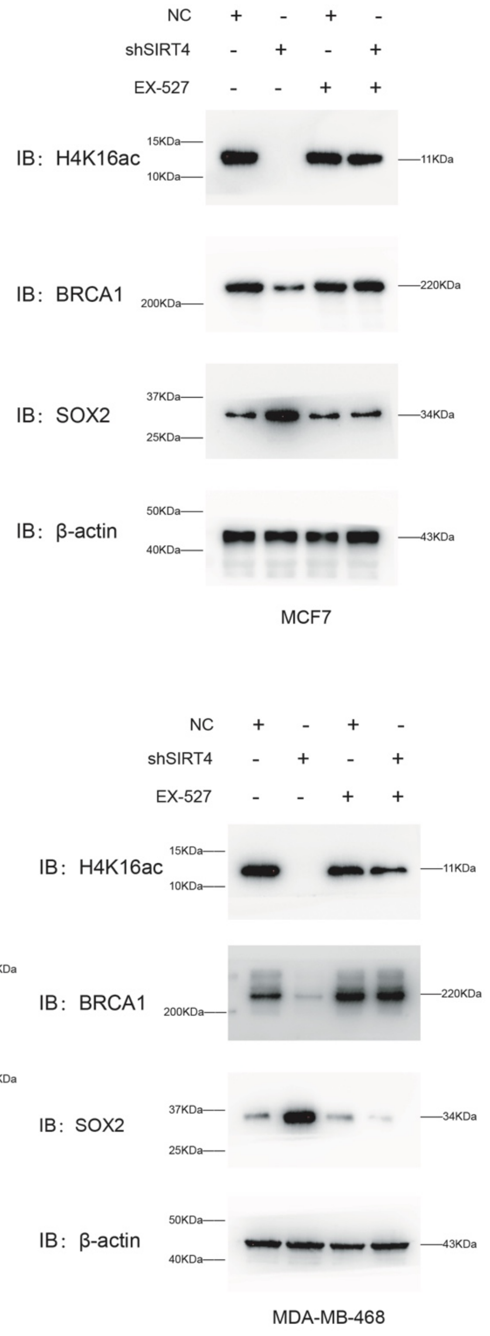

sFig6

A

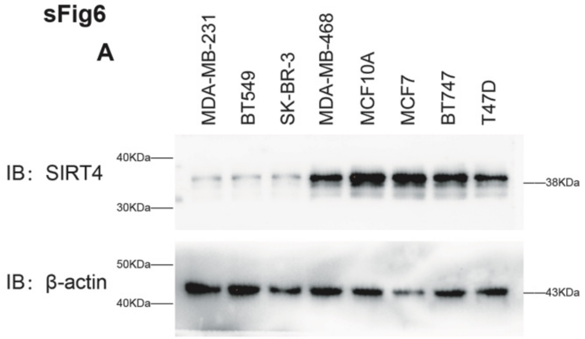

sFig 8

C

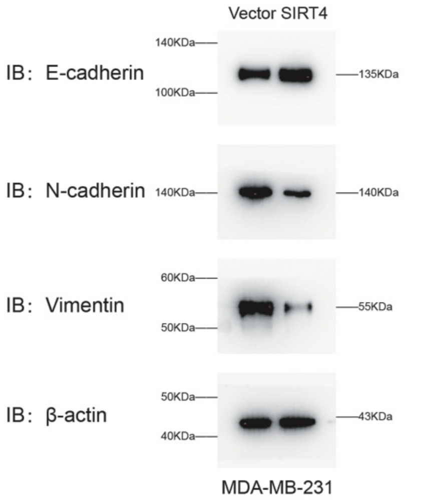

D

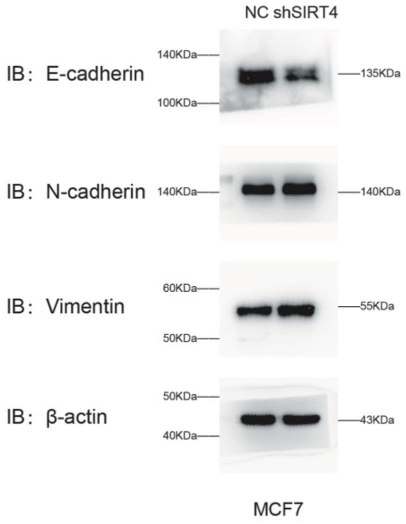

sFig 9

A

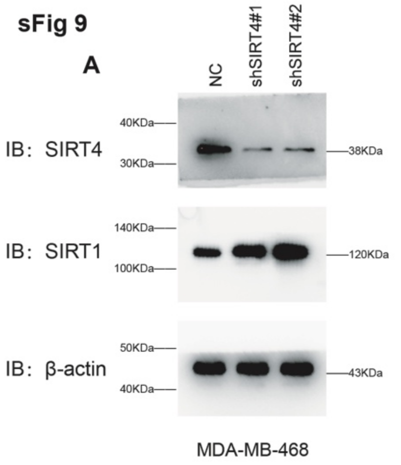

C

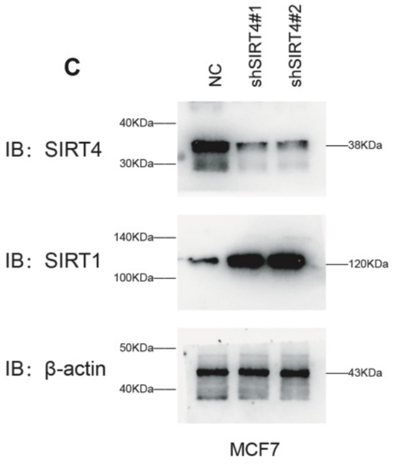

sFig 12

B

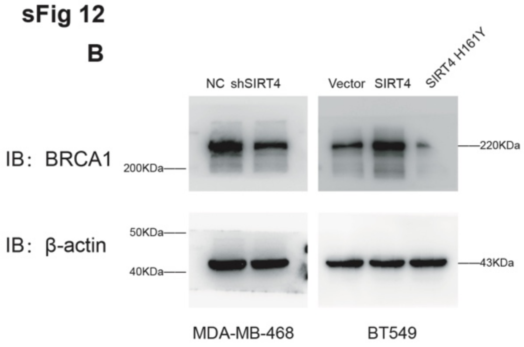

sFig 11

A

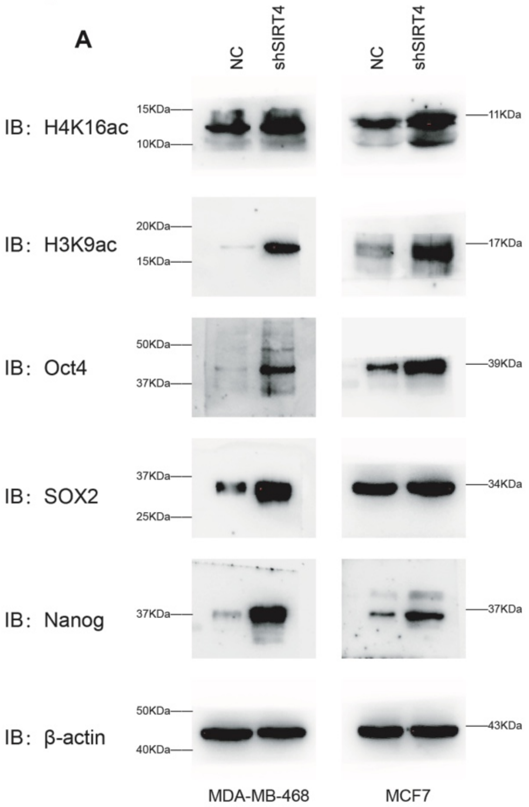

sFig 15

A

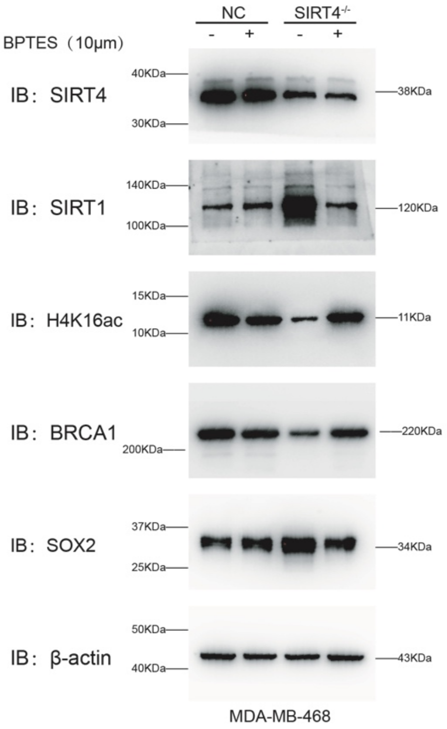

E

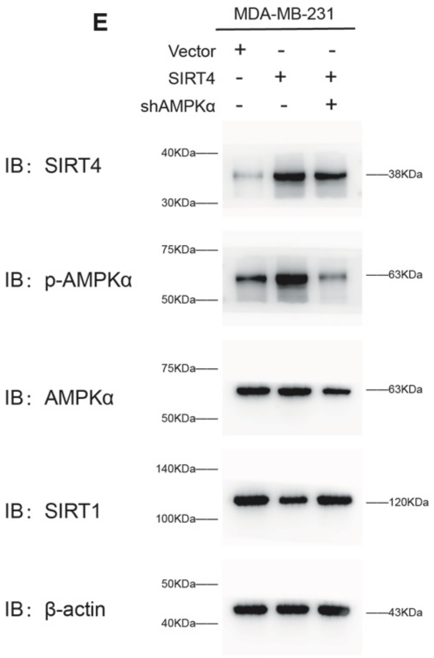

F

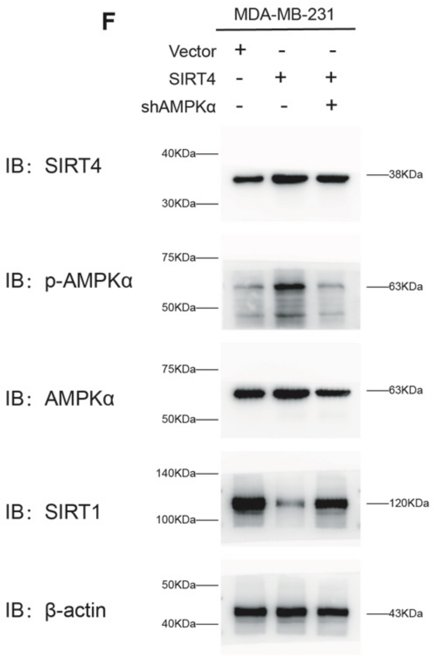

Supplement: Supplementary file 1 — Supplementary figures. [file thnov10p9458s1.pdf]
